# Predictors of slow clinical response and extended treatment in patients with extra-pulmonary tuberculosis in Pakistan, A hospital-based prospective study

**DOI:** 10.1371/journal.pone.0259801

**Published:** 2021-11-12

**Authors:** Atiqa Ambreen, Sabira Tahseen, Ahmad Wali, Muhammad Jamil, Syed Zeeshan Haider Naqvi, Nauman Safdar, Tehmina Mustafa

**Affiliations:** 1 Department of Microbiology, Gulab Devi Hospital, Lahore, Pakistan; 2 Institute of Molecular Biology and Biotechnology (IMBB), The University of Lahore, Defence Road Campus, Lahore, Pakistan; 3 National TB Reference Laboratory, National TB Control Program, Islamabad, Pakistan; 4 The Centre for International Health, Department of Global Public Health and Primary Care, University of Bergen, Bergen, Norway; 5 Department of Tuberculosis and Chest Medicine, Gulab Devi Hospital, Lahore, Pakistan; 6 Social and Health Inequalities Network (SHINe), a not for Profit, Non-Government Organization, Sindh, Pakistan; 7 Department of Thoracic medicine, Haukeland University Hospital, Bergen, Norway; Indian Institute of Technology Delhi, INDIA

## Abstract

The optimal duration of treatment in different forms of extrapulmonary tuberculosis (EPTB) is not clearly defined. This study aimed to identify predictors of slow clinical response and extended anti-TB treatment in EPTB patients. Socio-demographic, clinical, and microbiological characteristics of EPTB patients registered for anti-TB treatment at a tertiary care hospital, were analysed for identification of predictors of extended treatment. A total of 251 patients (137 lymphadenitis, and 114 pleuritis) were included in the analysis. Treatment was extended to more than 6 months in 58/251 (23%) patients. In the multivariate regression analysis, culture-positive EPTB (*p* = 0.007) [OR (95% CI) = 3.81 (1.43, 10.11)], history of diabetes (*p* = 0.014) [OR (95% CI) = 25.18 (1.94, 325.83)], smokeless tobacco use (*p* = 0.002) [OR (95% CI) = 17.69 (2.80, 111.72)], and slow regression of local signs and symptoms after 2 months of treatment (*p* < 0.001) [OR (95% CI) = 17.09 [(5.79, 50.39)] were seen to be significantly associated with treatment extension. Identification of predictors of extended treatment can help clinical decisions regarding optimal duration of treatment. Further studies are needed to identify subgroups of EPTB patients who can benefit from a shorter or longer treatment regimen.

## Introduction

Tuberculosis (TB) is a global public health threat, accounting for 1.4 million deaths worldwide in 2019 [1]. Extra-pulmonary TB (EPTB) represented 16% of the 7.1 million incident cases that were notified in 2019, ranging from 8% in the World Health Organization (WHO) Western Pacific Region to 24% in the Eastern Mediterranean Region [2]. In Pakistan, EPTB represented about 19% of 334754 notified TB cases in 2019 [3]. It was demonstrated in the 1970’s and 1980’s that a combination of isoniazid, rifampin, and pyrazinamide can reduce the duration of treatment in TB patients from 18 months to 6 months [4]. Ever since the treatment of TB has been a ‘one-size-fits-all’ paradigm, with a 6-month regimen for all forms of drug-susceptible pulmonary TB (PTB) and most forms of EPTB, comprising of a 2-months intensive phase with 4 drugs (isoniazid, rifampin, pyrazinamide, and ethambutol) followed by a 4-months continuation phase with 2 drugs (isoniazid and rifampin) [5]. It has been shown that TB patients with severe disease require longer treatment and are at a higher risk for relapse following treatment [6, 7]. Standardised treatment approach may lead to undertreatment of some patients, resulting in non-sterilizing cure or increased risk of relapse [8, 9]. At the other end of spectrum, some TB patients with minimal disease may be given unnecessarily long treatment with potential toxicities in whom cure is potentially possible with shorter treatment [10]. The published evidence indicates that different duration of treatment may be needed to treat TB patients with different clinical presentations [11]. The recommendations for the duration of treatment for EPTB are not based on studies as robust as those for PTB [12, 13]. There are limited studies on the optimal duration of treatment in various forms of EPTB. For monitoring response to treatment WHO guidelines only address bacteriologically positive PTB, whereas there is no clear guidance for monitoring response to treatment among smear-negative PTB and EPTB patients or on predictors associated with unfavourable TB treatment outcomes [5]. The objective of our study was to identify the risk factors associated with delayed response in EPTB patients requiring extended treatment.

## Methods

### Study setting

The study was conducted at Gulab Devi Hospital (GDH), a tertiary care not-for-profit private facility located in Lahore, Pakistan, where a large number of presumptive TB patients visit for specialized TB care. More than 6000 TB cases including 21% EPTB are registered annually for treatment under directly observed treatment short-course (DOTS) program (unpublished GDH data 2011–2020, reported to TB control program). Many patients after establishing the diagnosis are also referred to TB clinics close to their residence for treatment.

### Study design

This was an observational prospective cohort study embedded in a large research project which aimed at improving diagnosis the of EPTB. Patients of all ages attending GDH’s chest out-patients department with presumptive TB in pleura or lymph nodes were invited to participate in the study. Patients who provided signed informed consent having no prior history of TB treatment were enrolled from April 2016 to August 2017.

Patients were first examined by the physician and diagnostic laboratory tests were carried out on the relevant extra-pulmonary samples. For patients presenting with enlarged lymph nodes, an excision biopsy was performed, and the sample was divided into two parts and sent for histopathology in formalin and microbiological examination in normal saline. For patients with pleural effusions, aspirated fluid was sent for cytology and microbiological workup. The specimens were processed for smear examination, Xpert MTB/RIF assay (Xpert), and culture for detection of *Mycobacterium tuberculosis* on solid and automated liquid media [14]. Xpert was performed according to the manufacturer’s protocols [15]. Two slopes of Lowenstein-Jensen (LJ) medium and one Mycobacteria Growth Indicator Tube (MGIT^TM^ 960^TM^; Becton Dickinson, Sparks, MD, USA) were inoculated for culture [14]. All culture-positive cases were shipped to the National TB reference laboratory Islamabad, where they were processed for phenotypic drug susceptibility testing [16]. Patients were categorized as EPTB or non-TB patients. EPTB patients based on their convenience and preference to get treatment from GDH were registered for anti-TB treatment under the DOTS program as per WHO and NTP Pakistan guidelines [5, 17]. All patients were initiated on a standard 6-month anti-TB treatment including isoniazid, rifampin, pyrazinamide, and ethambutol for the first 2 months, followed by isoniazid and rifampin for 4 months. Patients were evaluated clinically by a physician at the end of 2 months of intensive treatment and again on completion of 6 months of treatment. At each follow-up visit response to treatment was assessed by 1) regression of symptoms, 2) regression of local signs of disease; regression of lymph nodes among lymphadenitis cases and regression of pleural effusion assessed by ultrasound among the pleuritis cases, 3) weight gain.

Based on clinical assessment patients were categorised into 1) responders, patients showing good clinical response, and 2) partial responders, patients showing some improvement but there was persistence of clinical signs and symptoms. After follow-up at 2-month, the continuation phase (isoniazid and rifampicin) was started in both groups. Treatment was completed at 6 months in patients showing complete regression of local signs and symptoms (responders), while it was extended beyond 6 months in partial responders. Patients with extended treatment were called every 2 months till complete regression of clinical signs and symptoms and treatment was then declared completed by the physician. Patients were excluded from the analysis if, a) there were unfavourable treatment outcomes (died, treatment failure, lost to follow-up) or, b) clinical evaluation was not done or not reported at 6 months of treatment.

All participants included in the study completed the EQ-5D-3L translated in the local language (Urdu version) facilitated by a trained health worker in a face-to-face interview at the TB clinic [18]. The translated version contained 4 questions related to healthcare; mobility, ability to perform usual activities, presence of pain/discomfort, and anxiety/depression.

The study protocol was approved by the Institutional Review Board, GDH, Lahore, National Bioethics Committee of Pakistan (Islamabad, Pakistan), and Regional Committee for Medical and Health Research Ethics, Western-Norway (REK Vest).

### Definition of patient groups and variables

Patients with a favourable outcome were divided into two groups based on the duration of treatment. ‘Patients with standard treatment duration’ were defined as EPTB patients treated with standard anti-TB treatment for 6 months, whereas, ’patients with extended treatment ‘ were defined as EPTB patients treated with standard anti-TB treatment for more than 6 months. Continuous variables like age, diagnostic delays, and duration of symptoms were dichotomised by using the median values as reference. Patients were divided into 2 groups according to BMI (<18.5 and ≥ 18.5). Bacterial load was estimated by culture results, culture-positive were considered as having high bacterial load, and culture-negative cases were considered as having a low bacterial load. Patients were categorised as diabetics if there were a history of diabetes or random blood sugar levels > 200 mg/dl at the time of inclusion in the study. Responses to the EQ-5D-3L questionnaire reported problems at three levels in each dimension and were described as a single index score for each patient. Patients were divided into 2 groups, i) no disability (level1), ii) some or extreme disability (level 2 or 3) [18]. Socioeconomic class status was calculated (based on the participant or parent’s/ guardian’s education level, main occupation, and monthly income), and divided into groups using the updated Kuppaswamy scale [19].

### Statistical analysis

The data were entered into Statistical Package for Social Sciences (SPSS) version 20 and cleaned for further analysis.

Exploratory analysis of predictors for extended treatment was performed by comparing patient characteristics between the two groups. Factors selected a priori were tested by binary (univariable) logistic regression to identify the frequency of each of the measured patient characteristics in each of the two groups and to identify those associated with extended TB treatment. Variables that showed a significant association (*p* < 0.05) in the binary regression analysis were introduced in the multivariable logistic regression model. Two variables (BMI and duration of constitutional symptoms) had more than 20% missing data and they were not included in multivariate regression analysis.

## Results

**Fig 1:**
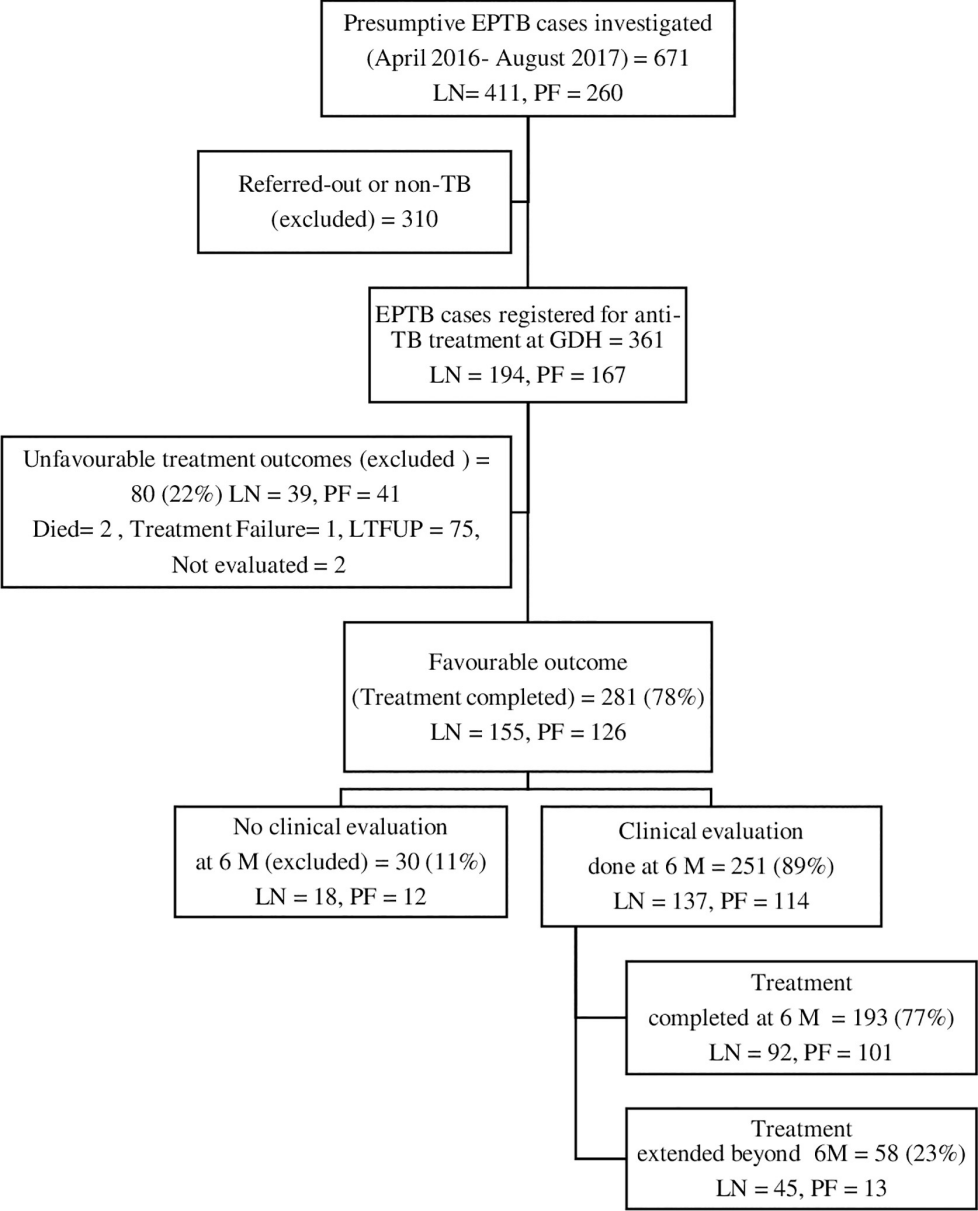
Flow chart showing the participants included in the analysis. LN: TB lymphadenitis, PF: TB pleuritis, EPTB: extrapulmonary tuberculosis, TB: tuberculosis, GDH: Gulab Devi Hospital, LTFUP: lost to follow-up, M: month.

Fig 1 shows the total number of registered EPTB patients and their treatment outcomes. Out of the 361 patients registered for treatment, a favourable outcome was recorded for 281 (78%) patients. The six months follow-up evaluation was available for 251/281 (89%) patients (137 TB lymphadenitis and 114 TB pleuritis). Standard treatment for 6 months was given to 193/251 (77%) patients, while in 23% (n = 58) of patients treatment was extended.

### Factors associated with extended treatment

Table 1 shows the results of the univariable regression analysis of different characteristics between the two groups. It was seen that, young age (median = 21 years) (*p* = 0.048) [OR (95% CI) = 1.83 (1.00, 3.34)], female gender (*p* = 0.025) [OR (95% CI) = 1.99 (1.09, 3.63)] and low BMI were significantly associated with treatment extension (*p* = 0.002) [OR (95% CI) = 3.68 (1.60, 8.49)].

**Table 1:** Analysis of patient and disease related characteristics associated with prolonged treatment by univariable logistic regression model.

	Standard treatment (6M) n/N (%)	Extended Treatment n/N (%)	OR	95% CI	p-value
**Age**					
> 21 years	102/193 (53)	22/58 (38)	1.00		
≤ 21 years	91/193 (47)	36/58 (62)	1.83	1.00–3.34	0.048
**Gender**					
Male	106/193 (55)	22/58 (38)	1.00		
Female	87/193 (45)	36/58 (62)	1.99	1.09–3.63	0.025
**Body Mass Index**					
≥ 18.5	77/135 (57)	9/34 (26)	1.00		
< 18.5	58/135 (43)	25/34 (74)	3.68	1.60–8.49	0.002
**Socioeconomic status** ^**a**^					
Level 2 (Upper Middle)	3/166 (2)	0/49 (0)	1.00		
Level 3 (Lower Middle)	64/166 (38)	14/49 (29)	0.00	0.00	0.999
Level 4 (Upper Lower)	99/166 (60)	35/49 (71)	0.61	0.30–1.24	0.176
**History of tobacco chewing**					
No	157/165 (95)	42/49 (86)	1.00		
Yes	8/165 (5)	7/49 (14)	3.27	1.12–9.53	0.030
**History of smoking**					
No	147/165 (89)	45/49 (92)	1.00		
Yes	18/165 (11)	4/49 (8)	0.72	0.23–2.25	0.580
**Diagnostic delays**					
≤ 69 days	105/190 (55)	22/57 (39)	1.00		
> 69 days	85/190 (45)	35/57(61)	1.96	1.07–3.59	0.029
**Co-morbid conditions** ^**b**^					
No	165/166 (99)	46/49 (94)	1.00		
yes	1/166 (1)	3/49 (6)	10.76	1.09–105.9	0.042
**Diabetes**					
No	186/190 (98)	49/54 (91)	1.00		
yes	4/190 (2)	5/54 (9)	4.74	1.22–18.33	0.024
**EQ-5D- 3L**					
Level 1^c^	27/155 (17)	3/46 (7)	1.00		
Level 2 or 3^d^	128/155 (83)	43/46 (93)	3.02	0.87–10.46	0.081
**Bacterial Load**					
Culture negative	121/193 (53)	25/58 (36)	1.00		
Culture positive	72/193 (47)	37/58(64)	2.21	1.22–4.02	0.009
**EPTB site**					
Pleura	101/193 (52)	13/58 (22)	1.00		
Lymph nodes	92/193 (48)	45/58 (78)	3.80	1.92–7.49	< 0.001
**Lymph nodes**					
Unilateral	89/92 (97)	44/44(100)	1		
Bilateral	3/92 (3)	0/44(0)	0.00	0.00	0.999
Matted/painful/discharging					
No	35/72 (49)	22/34 (65)	1		
Yes	37/72 (51)	12/34 (35)	0.51	0.22–1.19	0.123
**Associated pulmonary TB**					
No	159/163 (98)	42/ 43 (98)	1		
Yes	4/163 (2)	1/43 (2)	0.94	0.10–8.69	0.961
**Follow-up evaluations**					
**Clinical status at 2 months**					
Responders	142/193 (74)	12/58 (21)	1.00		
Partial responders	51/193 (26)	46/58 (79)	10.67	5.24–21.73	< 0.001
**Weight gain at 2 months**					
Any weight gain	122/183 (67)	31/56 (55)	1.00		
No weight gain	61/183 (33)	25/56 (45)	1.61	0.87–2.96	0.125
**Weight gain at 6 months**					
Any weight gain	165/183 (90)	42/53 (79)	1.00		
No weight gain	18/183 (10)	11/53 (21)	2.40	1.05–5.46	0.037

n: number of patients, N: total number of patients, OR: odds ratio, CI: confidence interval

^**a**^None of the patients fell in upper (level 1) and lower (level 5) socioeconomic class

^b^History of hypertension, renal or liver disease, level 1

^c^: no problem, level 2 and 3

^d^: some or extreme problems.

History of tobacco chewing (*p* = 0.030) [OR (95% CI) = 3.27 (1.12, 9.53)], presence of co-morbid conditions (history of liver disease, renal disease, hypertension) (*p* = 0.042) [OR (95% CI) = 10.76 (1.09, 105.9)], and diabetes (*p* = 0.024) [OR (95% CI) = 4.74 (1.22, 18.33)] were shown to be associated with slow response requiring extension of anti-TB treatment. More patients with diagnostic delays (median 69 days) (*p* = 0.029) [OR (95% CI) = 1.96 (1.07, 3.59)] required extended treatment as compared to the patients who received timely diagnosis and medical care.

More patients with culture-positive EPTB required treatment extension as compared to culture-negative cases indicating that bacterial load is an important determinant of treatment duration (*p* = 0.009) [OR (95% CI) = 2.21 (1.22, 4.02)]. TB lymphadenitis patients were seen to have more extension as compared to TB pleuritis patients (*p* < 0.001) [OR (95% CI) = 3.80 (1.92, 7.49). In our cohort of TB lymphadenitis patients, only 3/136 (2%) patients had bilateral lymph nodes and this was not found to be associated with the extension of treatment. Similarly, the presence of tender, matted, or discharging lymph nodes or the presence of PTB on chest X-ray did not seem to affect treatment duration.

The majority of patients who showed a good clinical response (142/154, 92%) at 2 months of treatment did not require treatment extension, while a higher proportion of patients in whom symptoms did not regress completely by 2 months required extended treatment (p < 0.001) [OR (95% CI) = 10.67 (5.24, 21.73)]. Lack of weight gain alone at 2 months was not an indicator of treatment extension, however weight gain alone at 6 months could be used to predict good response and an indicator of completion of treatment at 6 months, as no weight gain at 6 months was significantly associated with treatment extension (*p* = 0.037) [OR (95% CI) = 2.40 (1.05, 5.46)].

### Association of constitutional symptoms with extended treatment

Table 2 shows that longer duration of constitutional symptoms (> 8 weeks) was seen to be significantly associated with an extension of treatment (*p* = 0.004) [OR (95% CI) = 2.90 (1.40, 6.00)]. When individual symptoms and their duration were analysed, only the history of fever of more than 4 weeks was associated with treatment extension (*p* = 0.001) [OR (95% CI) = 3.66 (1.66, 8.05)].

**Table 2:** Analysis of constitutional symptoms and duration of symptoms with prolonged treatment by univariable logistic regression model.

	Standard treatment (6M) n/N (%)	Extended Treatment n/N (%)	OR	95% CI		p-value
Constitutional symptoms						
No	11/166 (7)	5/49 (10)	1.00			
yes	155/166 (93)	44/49 (90)	0.62	0.20	1.89	0.405
Duration of constitutional Symptoms						
≤ 8 weeks	121/151 (80)	25/43 (58)	1.00			
> 8 weeks	30/151 (20)	18/43 (42)	2.90	1.40	6.00	0.004
**Individual Symptoms**						
Fever						
No	27/163 (17)	10/49 (20)	1.00			
Yes	136/163 (83)	39/49 (80)	0.77	0.34	1.73	0.535
Duration of fever						
≤ 4 weeks	79/130 (61)	11/37 (30)	1.00			
> 4 weeks	51/130 (39)	26/37 (70)	3.66	1.66	8.05	0.001
Weight loss						
No	87/161 (54)	29/49 (59)	1.00			
Yes	74/161 (46)	20/49 (41)	0.81	0.42	1.55	0.526
Duration of weight loss						
≤ 4 weeks	41/67 (61)	8/18 (44)	1.00			
> 4 weeks	26/67 (39)	10/18 (56)	1.97	0.68	5.64	0.206
Appetite loss						
No	75/159 (47)	27/45 (60)	1.00			
Yes	84/159 953)	18/45 (40)	0.59	0.30	1.16	0.131
Duration of appetite loss						
≤ 4 weeks	47/74 (64)	7/15 (47)	1.00			
> 4 weeks	27/74 (36)	8/15 (53)	1.96	0.71	5.39	0.189
Night sweats						
No	120/158 (76)	37/46 (80)	1.00			
Yes	38/158 (24)	9/46 (20)	0.76	0.34	1.73	0.526
Duration of night sweats						
≤ 4 weeks	26/33 (79)	5/9 (56)	1.00			
> 4 weeks	7/33 (21)	4/9 (44)	2.97	0.62	14.10	0.17
Fatigue						
No	52/158 (33)	15/47 (32)	1			
Yes	106/158 (67)	32/47 (68)	1.04	0.52	2.10	0.898
Duration of fatigue						
≤ 4 weeks	70/100 (70)	16/29 (55)	1.00			
> 4 weeks	30/100 (30)	13/29 (45)	1.89	0.81	4.42	0.13

n: number of patients, N: total number of patients, OR: odds ratio, CI: confidence interval.

### Multivariate regression analysis

Table 3 shows the results of the multivariable logistic regression analysis. When the potential confounder variables, age, gender, history of tobacco chewing, presence of co-morbid conditions, diabetes, diagnostic delays, bacterial load, EPTB site, clinical evaluation at 2 month, and lack of weight gain at 6 months were adjusted by multivariate regression analysis to assess the independent effect of each of the factor, culture-positive EPTB (*p* = 0.007) [OR (95% CI) = 3.81 (1.43, 10.11)], history of diabetes (*p* = 0.014) [OR (95% CI) = 25.18 (1.94, 325.83)], smokeless tobacco use (*p* = 0.002) [OR (95% CI) = 17.69 (2.80, 111.72)], and slow regression of local signs and symptoms after 2 months of treatment (*p* < 0.001) [OR (95% CI) = 17.09 [(5.79, 50.39)] appeared to be significantly associated with extension of treatment. BMI and duration of constitutional symptoms were not added in the multivariable logistic regression as more than 20% values were missing.

**Table 3:** Multivariable logistic regression model for predictors of extended TB treatment.

	Unadjusted OR (95% CI)	p-value	Adjusted OR (95% CI)	p-value
**Age**				
> 21 years	1.00		1.00	
≤ 21 years	1.83 (1.00–3.34)	0.048	1.94 (0.68–5.51)	0.213
**Gender**				
Male	1		1.00	
Female	1.99 (1.09–3.63)	0.025	2.28 (0.70–7.36)	0.167
**History of tobacco chewing**				
No	1.00		1.00	
Yes	3.27 (1.12–9.53)	0.030	17.69 (2.80–111.72)	0.002
**EPTB Site**				
Pleura	1.00		1.00	
Lymph nodes	3.80 (1.92–7.49)	< .001	1.50 (0.46–4.86)	0.492
**Bacterial Load**				
Culture negative	1.00		1.00	
Culture positive	2.21 (1.22–4.02)	0.009	3.81 (1.43–10.11)	0.007
**Co-morbid conditions** ^**a**^				
No	1.00		1.00	
yes	10.76 (1.09–105.9)	0.042	4.74 (0.008–2757.77)	0.687
**Diabetes**				
No	1.00		1.00	
yes	4.74 (1.22–18.33)	0.024	25.18 (1.94–325.83)	0.014
**Diagnostic delays**				
≤ 69 days	1.00		1.00	
> 69 days	1.96 (1.07–3.59)	0.029	2.72 (0.98–7.49)	0.053
**Clinical status at 2 months**				
Responders	1.00		1.00	
Partial responders	10.67 (5.24–21.73)	< 0.001	17.09 (5.79–50.39)	< 0.001
**Weight t gain at 6 months**				
Any weight gain	1.00		1.00	
No weight gain	2.40 (1.05–5.46)	0.037	0.61 (0.186–1.99)	0.414

OR: odds ratio, CI: confidence interval, EPTB: extrapulmonary tuberculosis

^a^History of hypertension, renal or liver disease.

## Discussion

There are some published reports on factors predicting unfavourable treatment outcomes in EPTB [20–22], but to the best of our knowledge, this is the first study to explore the factors affecting extension of treatment in EPTB patients in a high TB burden setting. Univariable logistic regression analysis showed that age ≤ 21 years, female gender, low BMI, lymph node TB, bacteriologically positive EPTB, tobacco chewing, presence of co-morbid conditions, diabetes, diagnostic delays of more than 69 days, presence of constitutional symptoms > 8 weeks, failure of regression of local signs at 2 months of treatment, and lack of weight gain at 6 months, were significantly associated with prolonged treatment. In multivariable regression analysis only 4 factors; culture positive EPTB, tobacco chewing, diabetes, and failure of regression of local signs at 2 months of treatment were found to be significantly associated with the possibility of treatment extension.

Different studies have addressed the severity of mycobacterial disease estimated by bacillary load (presence of viable bacteria) and treatment outcomes, and have shown that smear and culture-positive PTB patients with high bacillary load take longer to convert [23, 24]. It has been also documented that extended treatment reduces relapse in PTB patients with a high disease burden [25], while it is possible to manage PTB patients with a minimal disease with shorter drug regimens [26, 27]. There are no published reports on optimal treatment duration required for EPTB patients with a high bacterial burden. Our findings show that pre-treatment culture positivity is an important factor associated with treatment extension.

The end of the intensive phase is an important landmark in TB treatment. Clinical regression of signs and subjective improvement of symptoms after 2 months of treatment are important criteria indicating good clinical response in TB patients. While no single clinical parameter before anti-TB treatment can reliably predict treatment response, sputum smear and culture status after 2 months of anti-TB medication are the most commonly used indicators in PTB [28, 29]. However, endpoints to monitor treatment response in EPTB are not easy as one cannot access tissue repeatedly, leaving clinical evaluation an important tool in monitoring treatment response during treatment [30]. Our findings suggest that patients in whom local symptoms do not regress at 2 months are most likely to get an extension of treatment. Studies on the shortening of anti-TB treatment show that 4 months regimen may be enough for drug-susceptible PTB [26, 27, 31, 32], findings from our study could be used to suggest that the EPTB patients that show clinical regression at 2 months may be the candidates for short treatment regimen trials in future studies. It is important to identify patients who may be treated with shorter drug regimens as unnecessary prolonged treatment may increase the risk of disruption of the human microbiome and other adverse effects, in patients taking these medicines [33].

Diabetes is found to be an important risk factor affecting sputum conversion, and uncontrolled diabetes is reported to be an independent risk factor for poor treatment response in PTB [34, 35]. Delayed sputum conversion (PTB patients) and high TB treatment failure rates were reported in diabetic patients among PTB and EPTB patients [36–38]. The effect of diabetes on treatment duration in EPTB has not been explored before. Our results show that diabetes slows response to anti-TB drugs requiring an extension of treatment in EPTB patients.

A large number of studies have evaluated the risk of tobacco smoking with poor treatment outcomes in PTB [39, 40], resulting in powerful campaigns against cigarette smoking. Tobacco chewing (smokeless tobacco use) often goes undetected in TB patients. A study from India reported a higher prevalence of tobacco chewing as compared to tobacco smoking among PTB patients [41]. While treating TB patients’ history of smokeless tobacco use should be asked as these patients may require prolonged treatment.

We observed an association of low BMI at the time of presentation with prolongation of treatment. It has been reported in the literature that low BMI indicates unfavourable treatment outcomes among smear-negative PTB and EPTB patients [42, 43]. The association between weight change during anti-TB therapy and its effect on treatment duration in EPTB has not been well studied. Previous studies have shown that weight changes during early TB treatment can be useful indicators of TB treatment outcomes [44, 45]. We did not find an association of early weight gain (after 2 months of treatment) with duration of treatment, however, lack of weight gain after 6 months of treatment was seen to be associated with treatment extension on binary regression, but significance was not detectable on multivariable logistic regression.

Diagnosis of EPTB is often based on presumptive and circumstantial evidence in resource-limited settings, with the consequence of a possible misdiagnosis [46]. In routine TB control programmatic settings, treatment is often continued despite the absence of improvement indicating a misdiagnosis. Careful clinical evaluation of patients during follow-up visits and identification of patients with poor response needs to be done systematically in routine TB care. This will help to identify patients for referral for alternative diagnostic evaluation or the need to extend the duration of treatment beyond 6 months.

Our study has some limitations, i) there was missing data especially height of the patients failing to calculate BMI in all patients, ii) clinical evaluation was not available for all patients after 6 months of treatment as some patients collected medicines at 5 months and did not return for last follow-up evaluation at 6 months, iii) long term follow-up of patients was not done after completion of treatment and we do not know the long term fate of these patients, iv) we were able to include only TB pleuritis and TB lymphadenitis patients in our study as this study was nested in a large research project aimed at improving the diagnosis of EPTB by the implementation of a sensitive and specific assay in routine TB diagnosis, and only previously undiagnosed new EPTB cases where a sample could be obtained from the disease site were included in the study. During the study period, the hospital did not have the facility to obtain biopsy/samples from EPTB sites other than pleural fluid and lymph node TB. This study was done in a routine hospital implementation setting which is a strength as well as a limitation because we were not able to control many factors which can influence patients’ inclusion and follow-up. Further studies are needed with long-term follow-up of the patients after treatment completion. Our sample size was also not as large as we expected as many EPTB patients were referred to DOTS centres close to their residences for registration or were lost to follow-up. The results from this study need to be validated in larger patient populations as well as in different epidemiological settings.

## Conclusion

In routine clinical setups importance of EPTB is usually masked by PTB. The optimal duration of treatment in various forms of EPTB needs to be tailored according to the patients’ characteristics and treatment response. Identification of risk factors associated with treatment prolongation can help clinical decision towards the optimal duration of treatment, thereby reducing the risk of relapse. Further studies are needed with a shorter treatment duration on sub-groups of EPTB patients.

## Supporting information

S1 File Questionnaire examination adults (English). (DOCX)

S2 File Questionnaire examination adults (Urdu). (DOCX)

S3 File Questionnaire examination children (English). (DOCX)

S4 File Questionnaire examination children (Urdu). (DOCX)
