# Supplementary material for: Predictors of slow clinical response and extended treatment in patients with extra-pulmonary tuberculosis in Pakistan, A hospital-based prospective study
Source: PLoS One. 2021 Nov 12;16(11):e0259801. doi: 10.1371/journal.pone.0259801 (PMC8589173; doi:10.1371/journal.pone.0259801)
Supplement: S1 File — (DOCX) [file pone.0259801.s001.docx]

**STUDY: IMPROVED DIAGNOSIS OF EXTRAPULMONARY TB**

**PATIENT REGISTRATION FORM**

**QUESTIONNAIRE–15 years and above**

**Date:**

**Paramedic/ Doctors** (who is interviewing the patient)**:**

**Hospital: Gulab Devi Hospital**

**Department:** OPD  IPD

**Extrapulmonary TB Suspect:**  Yes  No

| **INFORMED CONSENT** |
| --- |

**Informed consent (Part 1)**

** Yes  No**

***If Yes, complete sections A-F below.***

| **SECTION- A**  **PATIENT IDENTIFICATION** |
| --- |

**Name of patient:** ____________________________ **Study Number(three digit e.g.000)**: _______

**Age (years):**________

**Gender:** Male  Female

**Respondent:**  Patient  Parent  Spouse  Child  Other, relative/friend

**Address:** District __________City____________Village/Street/House____________________

| **SECTION- B**  **PERSONAL INFORMATION** |
| --- |

1. **Marital status:**

 Single  Married  Widow/widower  Separated

 Divorced  Other, please specify­­­­­­­­­____________

1. **Level of education:**

 No formal education  Not completed primary school

 Completed primary school Completed middle school

 Completed secondary school Above secondary school

Adult education Others (Please mention)______

1. **Religion**:

Muslim  Christian  Other, please mention____________

1. **Do you use chewable tobacco** (e.g.Pan, Niswar, Gutka)**:** Yes  No ­­­____weeks/months /years
2. **Do you smoke cigarettes/ Huka (water pipe):** Yes  No ­­­____weeks/months /years

| **SECTION- C**  **PAST MEDICAL HISTORY** |
| --- |

1. **Do you have any of these diseases?**

**COPD:**  Yes  No

**Renal Disease:**  Yes  No

**Liver Diseases:**  Yes  No

**Diabetes Mellitus:**  Yes  No

**Hypertension:**  Yes  No

**Other:**  Yes  No

**Describe other: ­­­­­­­­­­­­­­**_____________________________

_____________________________

| **SECTION-D**  **HEALTH SEEKING BEHAVIOUR & DIAGNOSTIC DELAY** |
| --- |

***Health seeking behavior of TB patients***

***Please remind the patient that this survey is confidential.***

1. **Please ask if the patient has experienced any of the following symptoms**
   1. **General Symptoms**

**Fever:**  Yes  No ­­­____weeks/months

**What kind of fever do you have?**  High-grade  Low-grade

**When do you have fever?** Morning  Day-time  Evening  Night  all day

**Loss of weight:**   Yes  No ­­­____weeks/months

**Loss of appetite:**  Yes  No ­­­____weeks/months

**Night Sweat:**  Yes  No ­­­____weeks/months

**Fatigue:**  Yes  No ­­­____weeks/months

**Amenorrhea(female only):**  Yes  No ­­­____weeks/months

**Body weakness:**  Yes  No ­­­____weeks/months

**Frequent cold:**  Yes  No ­­­____weeks/months

**Neck mass:**  Yes  No ­­­____weeks/months

**Other:**  Yes  No ­­­____weeks/months

**Describe:_________________________**

- 1. **Respiratory Symptoms**

**Cough:**   Yes  No ___weeks/months

**Sputum:**  Yes  No ___weeks/months

**Cough with Sputum:**  Yes  No ___weeks/months

**Blood with Sputum:**  Yes  No ___weeks/months

**Chest pain:**  Yes  No ___weeks/months

**Difficulty in breathing:**  Yes  No ___weeks/months

- 1. **Abdominal Symptoms**

**Swelling of/in stomach:**  Yes  No ­­­ ___weeks/months

**Fullness of stomach:**  Yes  No ___weeks/months

**Vomiting:**  Yes  No ___weeks/months

**Diarrhea:**  Yes  No ___weeks/months

**Other:**  Yes  No ___weeks/months

**Describe other: ­­­­­­­­­­­­­­**____________________________________________

____________________________________________

- 1. **Neurological Symptoms**

**Headache:**  Yes  No ___weeks/months

**Photophobia:**  Yes  No ___weeks/months

**Vomiting:**  Yes  No ___weeks/months

**Dizziness:**  Yes  No ___weeks/months

**Vertigo:**  Yes  No ___weeks/months

**Weakness/Numbness of extremity:**  Yes  No ___weeks/months

**Visual disturbance:**  Yes  No ___weeks/months

**Other:**  Yes  No ___weeks/months

**Describe other: ­­­­­­­­­­­­­­**_____________________________

_____________________________

_____________________________

1. **What were the major symptoms that first made you seek care?**

 Prolong Cough  Blood with sputum  Breathlessness

 Chest pain  Fever  Weight loss

 Fatigue\Weakness  Loss of appetite  Night sweats

Bone pain  Lymph node swelling  Diarrhoea

 Abdominal pain  others (specify) _________________

1. **When did you first notice the symptoms?**

______________________________________________________________________

1. **Did you practice any self-medication before you sought care?**  Yes  No
2. **How long did you experience these symptoms before you went to seek treatment?**

_____________________ (days/ weeks)

1. **How many different places did you go to seek help for the current symptoms? Number?______________and type of places?_________________**
2. **How many times have you visited health facilities with the same symptoms before?**

 First visit  Second visit  Third visit

 > 3 visits  don’t remember

1. **Which place did you first seek care for your symptoms?**

 Tertiary Care Hospital  District hospital  Rural health center

 Private Hospital/clinic  Traditional healer  Pharmacy

 other, please specify___________________________________

1. **What kind of diagnosis did you receive for your illness**? _______________________
2. **Were any tests done at the first medical service?**

 Yes  No

1. **What type of tests?**

 Blood test  Urine test  Sputum  X-ray

Others, please specify_______________________________

1. **Did you take the results back to the doctor?**

 Yes  No

1. **Could you estimate the total cost for the previous visits/investigations related to your current illness?**

Admission _________________________PKR

Consultations _________________________ PKR

Medication _________________________PKR

Laboratory tests/X-ray/CT________________________PKR

Transportation _________________________PKR

1. **Who has referred you to Gulab Devi Hospital?**

Self Traditional healers Religious leaders

Pharmacy/drug shop Village health worker Government dispensary

Government health center Government hospital Private dispensary/hospital

Charitable/NGO Member of the family Other___________________

1. **Before today, had you heard of the disease tuberculosis?**  Yes  No

**Pulmonary TB:**  Yes  No

**Extra pulmonary TB:**  Yes  No

1. **Do you have any one in your family who has been diagnosed with TB before?**

 Yes  No

If yes? From where he/she has taken treatment? ____________________________

1. **Before today, had you heard of the illness tuberculosis?**

 Yes  No

1. **Do you know any symptoms of tuberculosis?**

 Chronic cough  Spitting blood  Shortness of breath

 Chest pain  Fever Weight loss

 Tiredness  Loss of appetite  Lymph nodes enlargement

 Others Please specify_____________________________***(Do not probe but askfor more symptoms)***

1. **Do you know which parts of the body that can be affected by tuberculosis?**

**____________________________________________________________________**

1. **Can tuberculosis spread from person to person?**

 Yes  No  Uncertain

1. **Do you drink unboiled milk?**

 Yes  No

1. **Do people in your community stigmatize/ discriminate person having tuberculosis?**

 Yes  No  Uncertain

If yes, why? _________________________________

1. **Is there anything that would make it easier for people with tuberculosis to get treatment,**

**not just in this clinic, but in other health facilities?**

 Yes  No  Uncertain

If yes, what could be done? ___________________________________________

1. **What fears do others have about TB that prevents them from seeking medical advice?**

__________________________________________________________________

| **SECTION- E**  **EXAMINATION** |
| --- |

1. **Physical signs**
   1. **General**

**Weight:** ______K.g.

**Temperature:** ______ Deg. Centigrade

**Pulse rate**: ______b.p.m

**Blood pressure:** __________

**Pallor:**  Yes  No

**Finger clubbing:**  Yes  No

**BCG scar:**  Yes  No

**Other:**  Yes  No

- 1. **Lymph nodes**

**Lymph node enlargement:**  Yes  No

**Matted:**  Yes  No

**Painful:**  Yes  No

**Discharge/Sinus:**  Yes  No

**Please draw enlarged lymph nodes or other findings:**


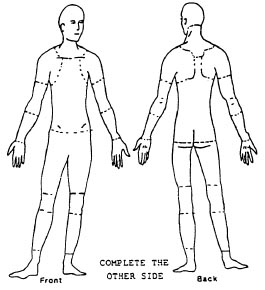


- 1. **Other Clinical Findings (as documented by the physicians/specialist)___________________**

**___________________________________________________________________________________**

| **SECTION- F**  **INVESTIGATIONS** |
| --- |

1. **Blood sample**

Hb ______________ ESR _____________

White blood cell count: ____________________________________________________________

1. **Bacteriology result**
   1. **Sputum examination**

**AFB microscopy**

**Date (day.month.year) Appearance * Neg. + ++ +++**

**¯¯¯¯¯¯¯¯¯¯¯¯¯¯¯¯¯¯¯¯¯¯¯¯¯¯¯¯¯¯¯¯¯¯¯¯¯¯¯¯¯¯¯¯¯¯¯¯¯¯¯¯¯¯¯¯¯¯¯¯¯¯¯¯¯¯¯¯¯¯¯¯¯¯¯¯¯¯¯¯¯¯**

**Sample 1 (Spot-1)________________________________________________________________**

**Sample 2 (Morning)______________________________________________________________**

*visual appearance (blood stained, muo-purulent, saliva)

**MTB Culture**  Positive Negative

Date of positive culture (day, month, year):________________________

**GeneXpert**   Positive  Negative

RIF resistant Yes  No

- 1. **Other samples investigated** (pleura fluid, ascites, lymph node biopsies, FNA, CSF)

**Material: _____________________________________**

Laboratory serial number: __________________

**AFB microscopy:**   Positive Negative

**Cytology/histology: __________________________________________________________**

**___________________________________________________________________________**

**MTB Culture**:  Positive Negative

**MTP64:**  Positive Negative

**Biochemical tests**: Protein _________ Glucose_________ Cell count: _________________

**GeneXpert:**  Positive  Negative

RIF res  No  Yes

**Gram stain:________________________________ Bact. Culture:____________________________**

**Other tests:** _________________________________________________________________________

1. **Other Investigations**
   1. X-Ray Chest

________________________________________________________________________

________________________________________________________________________

- 1. Sonography/CT scan

________________________________________________________________________

________________________________________________________________________

- 1. Any other (specify): ___________________**______________________________**

| **SECTION-G**  **PATIENT WHO ARE REGISTERED FOR EPTB TREATMENT & FOLLOW-UP** |
| --- |

**TB Registration Number (TB03):** _________________

**Final Diagnosis: _**________________________________

**Patient Condition at the time of follow-up** (Clinically improved, Not improved, Somewhat improved):

| Follow-up 1. | Follow-up 3. | Follow-up 5. |
| --- | --- | --- |
| Follow-up 2. | Follow-up 4. | Follow-up 6. |

| **SECTION- H**  **QUALITY OF LIFE** |
| --- |

1. **Quality of Life (at time of registration):**
   1. **Are you able to walk?**

 I have no problem in walking about  I have some problem in walking about  I am confined to bed

- 1. **Are you able to perform usual activity? (such as work, studies, domestic work, etc)**

 I have no problem with performing my usual activity  I have some problem with performing my usual activity  I am unable to perform my usual activity

- 1. **Are you having any pain/ discomfort?**

 I have no pain/discomfort  I have moderate pain/discomfort  I have extreme pain/discomfort

- 1. **Are you anxious/ depressed?**

 I am not anxious/ depressed  I am moderately anxious/ depressed  I am extremely anxious/ depressed

| **SECTION-I**  **PATIENT AND HOUSEHOLD COSTS** |
| --- |

***Estimate of the patient income level***

1. **How long does it take you to go to the nearest health facility?**

 Less than 30 minutes between 30 minutes and one hour More than one hour

1. **How far is this hospital to your home (in Kilometers) _________________**
2. **How long (on average) does it take you to this health facility, waiting for your**

**consultation and finally returning to your home\workplace?** ________________Hours

1. **How did you get to this health facility?**

 Walked  Bicycle Motorcycle  Private car  Rikshaw/taxi  Bus

1. **If you have to take a public transport (e.g. Rikshaw /taxi/ bus)how much (on average) does it cost you to come to the clinic? ________________PKR.**
2. **Do you have to make some special arrangements at home before coming to the Hospital? For example: To look after your children back home in your absence, any disabled persons, pregnant women or any job related arrangements?**

 Yes  No  Uncertain

If yes, what arrangements?­­­­­­­­­­­­­­­­­__________________________

1. **What is your main occupation (past twelve months)?**

 Employed by government  Employed private

 Self-employed (mention the self-employment such as: merchant/shop keeper/ farmer/ fishing/property agent/ etc)___________  Student  Housewife  Other_________________

1. **What is the main source of income of you and your house holds?**

 Employment (Govt or private)  Pensions

 Crop production  Livestock  Fishing

 Hunting / bee-keeping  Poultry  Farm wage

 Other agricultural activity  Wages (government)  Wages (private)

 Monetary savings (interest)  Property (rentals) Self-employed payments (merchant) Other Specify____________________________

1. **How much did (NAME) earn (money) for the activities stated on average in the past 12**

**months? This should include not only salary or cash income: but also the value of goods**

**produced or traded for other goods and services.**

**_____________________________________________________________________**

Between PKR:

 Less than 10,000

 10,000 – 20,000

 21,000-30,000

 31,000 – 40,000

 41,000-50,000

 More than 50,000

1. **Do you have reduced working capacity due to your current illness?**

 Yes, completely stopped working  Yes, working but with reduced capacity

 Working as normal

1. **Have you/or any member of your household lost any wages or income because of your illness?**

 Yes  No  Uncertain

If yes, how much____________________

1. **Do you own a house?**

 Yes renting a house  living with relatives /friends  Homeless

1. **How many people live in your household:** _________________ (number of people)
2. **What is the main source of drinking water for members of your household?**

 Piped water 1=Piped into dwelling 2= Piped into yard/plot 3=Public tap 4=Neighbors’ tap

 Hand Pump

 Water supplied by Tanker/Truck

 Water from open well

 Tube well/Turbine

 Running water 1=spring; 2=river/stream; 3=pond/Lake; 4=Dam

 Rain water

Water vendor

Bottled water

 Others Specify ________________________

1. **What kind of toilet facilities do members of your household usually use?**

 Flush to piped sewer system  Flush to septic tank

 Open Pit  Ventilated improved pit (VIP)  Public Latrine

 No facility/bush/field  other, please specify ______________

1. **Does your household have?**

 Electricity  Gas  Radio  Television  Telephone/mobile  Iron (either charcoal or electricity)  Refrigerator

1. **What is the main source of energy for lighting in your household?**

 Main electricity  Solar  Gas  Kerosene lamp

 Firewood  Candles  other, please specify _________________________

1. **What is the main material for the walls of your house or house you are living?**

 Mud  Cement bricks  Backed bricks  Wood

Stones  Others Specify_________________________

1. **What is the roofing material of your house or house you are living?**

 Grass/leaves/mud  Iron sheets  Tiles  Concrete/Cement

 Others Specify_______________

1. **Does you or any member of your household own?**

 A bicycle  A motorcycle or motor scooter  A car  A bank account

1. **How many acres of land for farming/grazing are owned by the household?**

 Arable land_____________acres  Land for grazing__________acres

1. **How many meals does your household usually have per day?**

Meals (in number)?_________________

**Informed consent (Part 2- for Blood Dry Spot)**

** Yes  No**

***If Yes, take the blood sample on paper and store as per the guidelines.***

| **SECTION- J**  **DIABETES SCREENING** |
| --- |

1. **Pre-diabetic (risk of getting diabetes)**
   1. **Do you have a mother or father or brother or sister and/or own child with diabetes?**

 Yes  No  Uncertain

If yes, who ____________________

- 1. **BMI of Participant [Use the BMI chart)? ___________ _____**
  2. **Has a doctor ever told you that you have high blood pressure, or given you medication for it?**

 Yes  No  Uncertain

- 1. **Nationality? ______________________**
  2. **Risk calculation (score to establish as pre-diabetic)?__________________**

**(Use Finnish Scoring chart)**

1. **Patient is known diabetic (from question 6)?**

 Yes  No ______ weeks/months/ years

If yes? Are you taking medication for diabetes?

 Yes  No If yes? Which medicines?______________

**If unknown diabetic?**

**Screen with Random Blood Glucose (using gluco-meter)**

**Result of RBG? ___________ (mg/dl)**

**If RBG** $\boldsymbol{\geq}$ **140-199 mg/dl , perform OGTT (offer 75mg of glucose dissolved in water and check blood sugar after 2 hours)**

**Result of PPBG (Post prandial blood glucose)? ___________ (mg/dl)**

***If PPBG <140 mg/dl (normal), if*** $\boldsymbol{\geq}$ **140-199 *mg/dl (pre-diabetic) and if*** $\boldsymbol{\geq}$ **200 mg/dl *refer the patient to physician/ Diabetes Specialist with patient results.***

| **SECTION- K**  **END OF TREATMENT** |
| --- |

1. **Quality of Life (at end of treatment duration):**
   1. **Are you able to walk?**

 I have no problem in walking about  I have some problem in walking about  I am confined to bed

- 1. **Are you able to perform usual activity? (such as work, studies, domestic work, etc)**

 I have no problem with performing my usual activity  I have some problem with performing my usual activity  I am unable to perform my usual activity

- 1. **Are you having any pain/ discomfort?**

 I have no pain/discomfort  I have moderate pain/discomfort  I have extreme pain/discomfort

- 1. **Are you anxious/ depressed?**

 I am not anxious/ depressed  I am moderately anxious/ depressed  I am extremely anxious/ depressed

1. **Response to treatment (at end of treatment duration):**
   1. **Presenting complaints (signs and symptoms)?**

 Settled  Somewhat settled  Not settled

- 1. **Treatment outcome?**

 Treatment completed  Lost to follow-up  Treatment Failure  Died  Not Evaluated
