# Supplementary material for: Predictors of slow clinical response and extended treatment in patients with extra-pulmonary tuberculosis in Pakistan, A hospital-based prospective study
Source: PLoS One. 2021 Nov 12;16(11):e0259801. doi: 10.1371/journal.pone.0259801 (PMC8589173; doi:10.1371/journal.pone.0259801)
Supplement: S2 File — (DOCX) [file pone.0259801.s002.docx]

تحقيق : بيرون پھيپھڑوں والى ٹى بى کى تشخيص ميں بہترى

*مريض کا رجسٹريشن فارم*

*سوالنامه: 15 اور اس سے زائد سال كے لوگوں كيلئے*

*تاريخ:*

*پيراميڈک / ڈاكٹر (جس نے مريض سے انٹرويو ليا)*

*هسپتال: گلاب ديوى ہسپتال*

*شعبہ:  او پى ڈى* *** آئى پى ڈى***

*ممکنہ بيرون پھيپھڑوں والى ٹى بى  ہاں  نہيں*

| *سوالنامہ / انٹرويو کے ليے رضامندى* |
| --- |

***كيا آپ اس سوالنامه/انٹرويو كيلئے رضامند ہيں (*حصه *اول)***

*** ہاں  نہيں***

***اگر جواب ہاں ميں ہو تو درج ذيل* A-F *حصوں كو مكمل كريں۔***

| **حصه ـ A**  مريض كى شناخت |
| --- |

*مريض كا نام :* ***____________________________ سٹڈى نمبر (تين ہندسے مثلا 000)___________________***

*عمر* *(سال)*: **______________**

*جنس*: *** لڑكا  لڑكى***

*جواب دينے وال*ا**: * مريض  والدين  بيوى/خاوند  بچہ  ديگر، رشته دار / دوست***

*پتہ:* ***ضلع ________________ شہر _____________ گاؤں/گلى/گھر_________________________***

| حصه ـB  *ذاتى معلومات* |
| --- |

*1۔ ازدواجى حيثيت*:

*** غير شادى شده  شادى شده  بيوه / رنڈوا *** *عليحدگى*

*** طلاق يافتہ  ديگر، وضاحت كريں __________________***

*2۔ تعليمى معيار*

*** رسمى تعليم نہيں لى  پرائمرى سكول مكمل نہيں كيا***

*** پرائمرى پاس  مڈل پاس***

*** ميٹرک پاس  ميٹرک سے اوپر***

*** تعليم بالغاں  ديگر (درج كريں)__________________________***

*3۔ مذہب*

*** مسلمان  عيسائى  ديگر كوئى، (درج كريں) ______________________***

*4۔ كيا آپ چبانے والا تمباكو استعمال كرتے هيں (مثلا پان، نسوار، گٹكا)*

*** ہاں  نہيں _____ہفتوں /مہينوں / سالوں سے***

*5۔ كيا آپ سگريٹ / حقہ پيتے هيں۔*

*** ہاں  نہيں _____ہفتوں /مہينوں / سالوں سے***

| حصہ ـ **C**  *ماضى کے طبى مسائل* |
| --- |

**6***۔ کيا آپ کو درج ذيل ميں سے کوئى بيمارى ہے؟*

COPD*:* *** ہاں  نہيں***

*گردوں کى بيمارى****:  ہاں  نہيں***

*ذيابيطس (شگر) کى بيمارى****:  ہاں  نہيں***

***بلڈ پريشر/ بلند فشار خون  ہاں  نہيں***

***ديگر  ہاں  نہيں***

***وضاحت کريں ___________________________***

***___________________________***

| *حصہ ـ* **D**  *علاج کے حصول کا رويہ اور تشخيص ميں تاخير* |
| --- |

**ٹى بى کے مريض کا علاج کے حصول کا رويہ**

**مريض کو بتائيں کہ اس سوالنامہ / انٹرويو کو پوشيدہ رکھا جائے گا۔**

**7ـ مريض سے پوچھيں کہ کيا اُس نے درج ذيل علامات کا سامنا کيا**

**7.1 عام علامات**

بخار: *** ہاں  نہيں _______ ہفتے / مہينے***

***آپ کو کيسا بخار چڑھتا ہے:  زيادہ شدت کا  کم شدت کا***

***آپ کو کس وقت بخار چڑھتا ہے:  صبح  دن  شام  رات  تمام دن***

***وزن ميں کمى  ہاں  نہيں _______ ہفتے / مہينے***

***بھوک نہ لگنا  ہاں  نہيں _______ ہفتے / مہينے***

***رات کو پسينہ  ہاں  نہيں _______ ہفتے / مہينے***

***تھکاوٹ  ہاں  نہيں _______ ہفتے / مہينے***

***حيض بند ہونا (صرف لڑکى کيلئے)  ہاں  نہيں _______ ہفتے / مہينے***

***جسمانى کمزورى  ہاں  نہيں _______ ہفتے / مہينے***

***سردى کا زيادہ لگنا  ہاں  نہيں _______ ہفتے / مہينے***

***گردن ميں غدود  ہاں  نہيں _______ ہفتے / مہينے***

***ديگر کوئى  ہاں  نہيں _______ ہفتے / مہينے***

***وضاحت کريں ______________________________***

**7.2 سانس (نظام تنفس) کى علامات**

***کھانسى  ہاں  نہيں _______ ہفتے / مہينے***

***بلغم  ہاں  نہيں _______ ہفتے / مہينے***

***کھانسى کے ساتھـ بلغم  ہاں  نہيں _______ ہفتے / مہينے***

***بلغم کے ساتھـ خون  ہاں  نہيں _______ ہفتے / مہينے***

***سينے ميں درد  ہاں  نہيں _______ ہفتے / مہينے***

***سانس لينے ميں تکليف  ہاں  نہيں _______ ہفتے / مہينے***

**7.3 پيٹ کى علامات**

***معدے پر/ميں سوجن  ہاں  نہيں _______ ہفتے / مہينے***

***معدہ بھرا ہونا  ہاں  نہيں _______ ہفتے / مہينے***

***قے/اُلٹى  ہاں  نہيں _______ ہفتے / مہينے***

***اسھال  ہاں  نہيں _______ ہفتے / مہينے***

***ديگر  ہاں  نہيں _______ ہفتے / مہينے***

***وضاحت کريں ________________________________***

**7.4 اعصابى علامات**

***سر درد  ہاں  نہيں _______ ہفتے / مہينے***

***روشنى سے ڈر  ہاں  نہيں _______ ہفتے / مہينے***

***قے/اُلٹى  ہاں  نہيں _______ ہفتے / مہينے***

***چکر  ہاں  نہيں _______ ہفتے / مہينے***

***نقاہت  ہاں  نہيں _______ ہفتے / مہينے***

***کمزورى/ سن ہونا  ہاں  نہيں _______ ہفتے / مہينے***

***نظر خراب ہونا  ہاں  نہيں _______ ہفتے / مہينے***

***ديگر  ہاں  نہيں _______ ہفتے / مہينے***

***وضاحت کريں ______________________________***

***______________________________***

***______________________________***

**8ـ وہ کونسى اہم علامات تھيں جن کى وجہ سے آپ علاج کى طرف متوجہ ہوئے**

*** طويل کھانسى  بلغم کے ساتھـ خون  سانس چڑھنا***

*** سينے ميں درد  بخار  وزن ميں کمى***

*** تھکاوٹ/کمزورى  بھوک نہ لگنا  رات کو پسينہ***

*** ہڈيوں ميں درد  لمف نوڈ سوجن  ڈائريا / اسھال***

*** پيٹ کا درد  ديگر (وضاحت کريں) ______________________***

**9ـ آپ نے پہلى مرتبہ کب يہ علامات محسوس کيں؟**

***_____________________________________________________________________________***

**01ـ کيا آپ نے علاج سے قبل اپنے طور پر ادويات استعمال کيں۔ * ہاں  نہيں***

**11ـ بيمارى کى علامات محسوس کرنے کے کتنے عرصے بعد آپ نے طبى مشورہ ليا؟**

***________________________ دن /***  *ہفتے*

**12ـ موجودہ علامات کا علاج کرانے کيلئے آپ کتنى جگہوں پر مدد کيلئے گئے؟**

***تعداد؟ _________________ اور جگہوں کى نوعيت؟_________________________***

**13ـ اس سے قبل ان علامات کے ساتھـ آپ نے کتنى مرتبہ مراکز صحت کا چکر لگايا ؟**

*** پہلا چکر  دوسرا چکر  تيسرا چکر***

*** تين سے زائد چکر  ياد نہيں***

**14ـ اپنى علامات کے علاج کيلئے آپ پہلى مرتبہ کہاں گئے؟**

*** درجہ سوم نگہداشتى ہسپتال (بڑا ہسپتال)  ضلعى ہسپتال  ديہى مرکز صحت***

*** نجى ہسپتال/کلينک  روايتى حکيم  فارميسى***

*** ديگر، وضاحت کريں _______________________________________***

**15ـ آپ کى تکليف/علامات سے کونسى بيمارىتشخيص ہوئى؟** __________________________________

**16ـ پہلى طبى ملاقات پر آپ کے کوئى ٹيسٹ کرائے گئے ؟**

*** ہاں  نہيں***

**17ـ کس نوعيت کے ٹيسٹ ہوئے؟**

*** خون کا ٹيسٹ  پيشاب کا ٹيسٹ  بلغم/تھوک کا ٹيسٹ  ايکسرے***

*** ديگر، وضاحت کريں _______________________________________***

**18ـ کيا آپ ڈاکٹر کے پاس ٹيسٹوں کى رپورٹ لے کر گئے؟**

*** ہاں  نہيں***

**19ـ کيا آپ اپنى موجودہ بيمارى سے متعلق گزشتہ چکر / تشخيص کے اخراجات کا اندازہ لگاسکتے ہيں؟**

***داخلہ ___________________ روپے***

***مشاورتى فيس ___________________ روپے***

***ادويات ___________________ روپے***

***ليبارٹرى ٹيسٹ/ايکسرے/سى ٹى ___________________ روپے***

***سفرى اخراجات ___________________ روپے***

**20ـ گلاب ديوى ہسپتال ميں علاج کيلئے کس نے بھيجا؟**

*** خود  روايتى حکيم  مذہبى رہنما***

*** فارميسى/ادويات کى دکان  ديہى ہيلتھـ ورکر  سرکارى ڈسپنسرى***

*** سرکارى مرکز صحت  سرکارى ہسپتال  نجى ڈسپنسرى/ہسپتال***

*** خيراتى مرکز/اين جى او  خاندان کے فرد  ديگر__________________***

**21ـ آج سے قبل کيا آپ نے تپ دق (ٹى بى) کے بارے ميں سنا تھا؟** * ہاں  نہيں*

***پھيپھڑوں والى ٹى بى  ہاں  نہيں***

***بيرون پھيپھڑوں والى ٹى بى  ہاں  نہيں***

**22ـ کيا اس سے قبل آپ کے خاندان ميں کسى کو ٹى بى کا مرض ہوا؟**

*** ہاں  نہيں***

***اگر ہاں؟ تو اس نے کہاں سے علاج کروايا؟ _______________________________________***

**23ـ کيا آپ تپ دق (ٹى بى) کی کسی علامات کے بارے میں جانتے ہیں؟**

*** طويل کھانسى  بلغم کے ساتھـ خون  سانس چڑھنا***

*** سينے ميں درد  بخار  وزن ميں کمى***

*** تھکاوٹ/کمزورى  بھوک نہ لگنا  لمف نوڈ سوجن***

*** ديگر (وضاحت کريں) ______________________***

***(تحقیق کی بجائے مزید علامات جاننے کے لئے مزید سوالات کریں؟)***

**24ـ کيا آپ جانتے ہیں کہ تپ دق (ٹى بى) کی وجہ سے آپ کے جسم کے کونسے حصے متاثرہو سکتے ہیں؟**

***____________________________________________________________________***

**25ـ کیا تپ دق (ٹى بى) ایک شخص سے دوسرے شخص کو لگ سکتی ہے؟**

*** ہاں  نہيں***

**26ـ کيا آپ دودھ کو اُبالے بغير پيتے ہيں؟**

*** ہاں  نہيں***

**27ـ کيا آپ کے خاندان/علاقہ ميں لوگ تپ دق (ٹى بى) کے مريض سے اچھا برتاؤ نہيں رکھتے؟**

*** ہاں  نہيں  اندازہ نہيں ہے***

***اگر ہاں؟ تو کيوں؟ _________________________________________***

**28ـ اس کلينک کے ساتھـ ساتھـ ديگر مراکز صحت ميں ايسا کيا کيا جائے جس سے تپ دق (ٹى بى) کے مريضوں کو علاج ميں آسانى ہو؟**

*** ہاں  نہيں  اندازہ نہيں ہے***

***اگر ہاں؟ تو کيا ہوسکتا ہے؟ ___________________________________________________***

**29ـ ٹى بى کے بارے ميں دوسرے لوگوں کو کس قسم کے خدشات لاحق ہيں جو اُنھيں طبى مشورے سے دور رکھتے ہيں ؟**

***_______________________________________________________________________***

| *حصہ ـ* **E**  *معائنہ* |
| --- |

**30ـ جسمانى علامات**

**30.1 عام علامات**

وزن _______ کلوگرام

حرارت _______ ڈگرى سينٹى گريڈ

نبض کى رفتار _______ دھڑکن فى منٹ

بلڈپريشر _______

رنگت *** ہاں  نہيں***

انگليوں کا ارتعاش *** ہاں  نہيں***

BCG نشان *** ہاں  نہيں***

ديگر *** ہاں  نہيں***

**30.2 موادي گلٹياں (لمف نوڈز)**

لمف نوڈز /موادى گلٹياں بڑى ہونا *** ہاں  نہيں***

الجھا ہوا *** ہاں  نہيں***

تکليف *** ہاں  نہيں***

سوراخ/ اخراج والا *** ہاں  نہيں***

***برائے مہربانى موادى گلٹيوں (لمف نوڈز) يا ديگر نتائج کى تصوير ميں نشاندہى کريں۔***


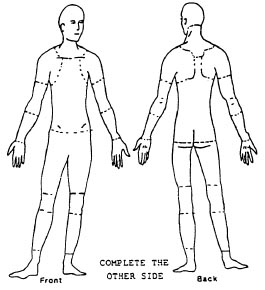


**30.3 ديگر طبى نتائج (جو ڈاکٹر/ماہر نے درج کيے ہوں)**____________________________________

________________________________________________________________________

| *حصہ ـ* **F**  *تحقيقات کے نتائج* |
| --- |

**31ـ خون کا نمونہ**

Hb__________________ ESR ________________

خون ميں سفيد خليوں کى تعداد _________________________________________________________

**32ـ بيکٹيريالوجى نتيجہ**

**32.1 بلغم کا تجزيہ**

AFBمائيکروسکوپى

تاريخ (سال ۔ ماہ ـ دن) شکل***** منفى۔ **+ ++ +++**

_____________________________________________________________________________

نمونہ 1: (سپاٹ ـ 1) _______________________________________________________________

نمونہ 2: (صبح) _________________________________________________________________

***** کس شکل ميں ہے (خون آلود، پيپ، تھوک)

**MTB کلچر**  *** مثبت  منفى***

***مثبت کلچر کى تاريخ (سال، ماہ، دن) ______________________***

**GeneXpert * مثبت  منفى***

RIF مزاحمت *** ہاں  نہيں***

**32.2 ديگر نمونے (پلورا فلوڈ، ايسيٹيس، لمف نوڈ بائيوسپيس، FNA، CSF)**

مواد: ___________________

ليبارٹرى سيريل نمبر: ___________

AFBمائيکروسکوپى *** مثبت  منفى***

***سائيٹالوجى/ہسٹالوجى _________________________________________________________***

***_______________________________________________________________________***

MTBکلچر: *** مثبت  منفى***

MTP64: *** مثبت  منفى***

بائيوکيميکل ٹيسٹ: پروٹين_____________ گلوکوز___________ خليون/ سيل کى تعداد ____________

GeneXpert: *** مثبت  منفى***

RIF res *** نہيں  ہاں***

***گرام سٹين ________________________________ بيکٹريل کلچر __________________________***

***ديگر ٹيسٹ ___________________________________________________________________***

**33ـ ديگر ٹيسٹ**

**33.1 ايکسرے چھاتى/سينہ**

***__________________________________________________________________________***

***__________________________________________________________________________***

**33.2 سونوگرافى / سى ٹى سکين**

***__________________________________________________________________________***

***__________________________________________________________________________***

**33.3 ديگر، وضاحت کريں،*______________________________________________________***

***__________________________________________________________________________***

| *حصہ ـ* **G**  *بيرون پھيپھڑوں والى ٹى بى کے علاج کیلیے مریض کی رجسٹریشن اور اس کا مرحلہ وار علاج* |
| --- |

**:(TB03)** *ٹی بی رجسٹریشن نمبر*

*مکمل تشخیص:*

*کے وقت مریض کی حالت :* **(Follow-up)***علاج مرحلہ وار*

*( طبی لحاظ سے بہتری آئی ہے،بلکل بہتری نہیں آئی، کچھ حد تک بہتری آئی ہے)*

| Follow-up 1. | Follow-up 3. | Follow-up 5. |
| --- | --- | --- |
| Follow-up 2. | Follow-up 4. | Follow-up 6. |

| *حصہ ـ* **H**  *مريض کی حالت* |
| --- |

**34***۔ رجسٹريشن كے وقت مريض کى حالت*

**34.1***۔ کيا آپ چلنے پھرنے کے قابل ہيں؟*

*** مجھے چلنے ميں كوئى مسئله نہيں ہے  مجھے چلنے ميں كچھـ مسئلہ ہے  ميں بستر تک محدود ہوں***

**34.2***۔ کيا آپ روزمرہ کام کرنے کے قابل ہيں؟ (جيسے کام، تعليم، گھريلو کام وغيرہ)*

*** مجھے روزمرہ کام کرنے ميں کوئى مشکل نہيں ہے  مجھے روزمرہ کام کرنے ميں تھوڑى مشکلات ہيں***

*** ميں روزمرہ کام کرنے کے قابل نہيں ہوں***

**34.3***۔ کيا آپ کو کسى درد / تکليف کا سامنا ہے ؟*

*** مجھے کوئى درد/تکليف نہيں ہے  مجھے تھوڑے درد/تکليف کا سامنا ہے  مجھے شديد درد/تکليف کا سامنا ہے***

**34.4***۔ کيا آپ اُداس / فکرمند ہيں؟*

*** ميں اُداس/فکرمند نہيں ہوں  ميں تھوڑا اُداس/فکرمند ہوں  ميں بہت اُداس/فکرمند ہوں***

| *حصہ ـ* **I**  *مريض اور گھريلو اخراجات* |
| --- |

*مريض کى آمدن کا اندازہ*

**35**۔ قريبى مرکز صحت جانے ميں آپ کا کتنا وقت لگتا ہے؟

*** 30 منٹ سے کم  آدھے سے ايک گھنٹہ  ايک گھنٹے سے زائد***

**36**۔ آپ کے گھر سے ہسپتال کا فاصلہ (کلوميٹرميں) کتنا ہے؟ **_______________________**

**37**۔ مرکز صحت آنے، مشاورت کيلئے اپنى بارى کے انتظار اور واپس گھر/کام پر پہنچنے ميں عموماً کتنا

وقت لگتا ہے؟ **____________ گھنٹے**

**38**۔ آپ مرکز صحت کس طرح آتے ہيں؟

*** پيدل  سائيکل  موٹرسائيکل  ذاتى گاڑى  رکشہ/ٹيکسى  بس***

**39**۔ کلينک آنے کيلئے پبلک ٹرانسپورٹ (مثلاً رکشہ/ٹيکسى/بس) استعمال کريں تو عموماً کتنى رقم

خرچ ہوتى ہے؟ **____________ روپے**

**40**۔ کيا آپ کو ہسپتال آنے سے پہلے گھر ميں کچھـ خصوصى انتظامات کرنے پڑتے ہيں؟ مثلاً آپ کى غيرموجودگى ميں بچوں، کسى معذور فرد يا حاملہ عورت کى ديکھـ بھال يا نوکرى سے متعلق کوئى انتظامات؟

*** ہاں  نہيں  اندازہ نہيں ہے***

***اگر ہاں؟ تو کيسے انتظامات؟ ___________________________________________________***

**41**۔ گزشتہ ايک سال سے آپ کا اصل پيشہ کيا ہے؟

*** سرکارى ملازم  پرائيويٹ ملازم***

*** اپنا کاروبار (اپنے کاروبار جيسے مرچنٹ/دکاندار/کسان/ماہى گيرى/پراپرٹى ايجنٹ وغيرہ درج کريں)_________***

*** طالبعلم  خاتون خانہ  ديگر ________________***

**42ـ آپ اور آپ کے گھرانے کا بنيادى ذريعہ آمدن کيا ہے؟**

*** ملازمت (سرکارى/پرائيويٹ)  پينشن***

*** فصلوں کى کاشت  مويشى پالنا  ماہى گيرى***

*** شکارکرنا  پولٹرى فارم  فارم پر اجرتى ملازم***

*** ديگر زرعى سرگرمياں  اجرت (سرکارى)  ديہاڑى (پرائيويٹ)***

*** مالياتى بچت (سود)  جائيداد کا کرايہ  اپنا کاروبار (دکاندار)***

*** ديگر__________________***

**43**۔ گزشتہ ايک سال ميں ان کاموں سے اوسط ماہانہ آمدن کيا ہے؟اس ميں صرف تنخواہ يا نقد آمدن ہى نہيں بلکہ پيداوار يا تجارتى اشياء کى قيمت اور خدمات بھى شامل کى جائيں۔

_____________________________________________________________________________

تقريباً پاکستانى روپے

*** 10000 سے کم***

*** 10000 سے 20000***

*** 21000 سے 30000***

*** 31000 سے 40000***

*** 41000 سے 50000***

*** تقريباً 50000 يا اس سے زيادہ***

**44**۔ کيا بيمارى کى وجہ سے آپ کے کام کرنے کى صلاحيت ميں کمى واقع ہوئى ہے؟

*** ہاں، ميرا کام مکمل طور پر بند ہوگيا ہے  ہاں، کام ہورہا ہے ليکن کم صلاحيت کے ساتھـ***

*** پہلے کى طرح کام کررہا ہوں***

**45**۔ کيا بيمارہونے سے آپ يا گھر کے کسى فرد نے تنخواہ يا آمدن ميں نقصان اٹھايا ؟

*** ہاں  نہيں  اندازہ نہيں ہے***

***اگر ہاں، تو کتنا؟_________________________***

**46**۔ کيا آپ کا رہائشى گھر اپنا ہے؟

*** ہاں  کرائے کا گھر  رشتہ دار / دوست کے ساتھـ رہائش  بے گھر***

**47**۔ آپ کے گھر ميں کتنے افراد رہائش پذير ہيں؟ ________________ (گھر کے افراد)

**48**۔ آپ کے گھر ميں پينے والے پانى کا بنيادى ذريعہ کيا ہے؟

*** پائپ لائين 1= گھر ميں پائپ لائين 2= صحن/پلاٹ ميں پائپ لائين 3= سرکارى نلکا 4= پڑوسيوں کا نلکا***

*** ہينڈ پمپ***

*** ٹينکر/ٹرک سے پانى کى فراہمى***

*** کھلے کنويں کا پانى***

*** ٹيوب ويل / ٹربائين***

*** چلتا پانى 1= چشمہ 2= دريا/ندى 3= تالاب/جھيل 4= ڈيم***

*** بارشى پانى***

*** پانى بيچنے والا***

*** بوتلوں کا پانى***

*** ديگر، وضاحت کريں ________________________________***

**49ـ عام طور پر آپ کے گھر والے کس قسم کا بيت الخلا/باتھـ روم استعمال کرتے ہيں؟**

*** پائپوں والا سيوريج سسٹم  سيپٹک ٹينک ميں نکاس***

*** کھلا گڑھا  ہوادار بہترين گڑھا***(VIP) *** عوامى بيت الخلا/ ليٹرين***

*** سہولت نہيں ہے/جھاڑياں/کھيت  ديگر، وضاحت کريں____________________***

**50ـ کيا آپ کے گھر ميں درج ذيل اشياء ہيں؟**

*** بجلى  گيس  ريڈيو  ٹيلى ويژن  فون/ موبائيل***

*** استرى (چاہے کوئلے يا بجلى والى)***

**51ـ آپ کے گھر ميں روشنى کيلئے توانائى کا بنيادى ذريعہ کيا ہے؟**

*** بجلى  شمسى توانائى  گيس  مٹى کے تيل والا ليمپ***

*** لکڑيوں کى آگ  موم بتى  ديگر، وضاحت کريں ______________________***

**52ـ آپ کے گھر يا رہائشى جگہ کى ديواريں کس چيز سے بنى ہوئى ہيں؟**

*** کيچڑ/گارا  سيمنٹ کى اينٹيں  پکى اينٹيں  لکڑى***

*** پتھر  ديگر، وضاحت کريں ______________________***

**53ـ آپ کے گھر يا رہائشى جگہ کى چھتيں کس چيز سے بنى ہوئى ہيں؟**

*** گھاس/پتے/کيچڑ  جستى چادريں  ٹائليں  کنکريٹ/سيمنٹ***

*** ديگر، وضاحت کريں ______________________***

**54ـ کيا آپ يا گھر کے کسى فرد کے پاس درج ذيل اشياء موجود ہيں؟**

*** سائيکل  موٹرسائيکل/سکوٹر  کار  بنک اکاؤنٹ***

*** ديگر، وضاحت کريں ______________________***

**55ـ آپ کے گھرانے کے پاس کاشتکارى/گلہ بنانى کيلئے کتنے ايکڑ زمين موجود ہے؟**

*** قابل کاشت زمين ________ ايکڑ  چرانے کيلئے زمين ________ ايکڑ***

**56ـ آپ کے گھروالے عام طور پر روزانہ کتنى مرتبہ کھانا کھاتے ہيں؟**

*** کھانوں کى تعداد (نمبروں ميں) _____________________***

*كيا آپ رضامند ہيں (*حصه *دوم) خشک خون کا نمونہ*

*** ہاں  نہيں***

| *حصہ ـ* **J**  *ذيابيطس سکريننگ* |
| --- |

**57ـ ابتدائى ذيابيطس (زيابيطس ہونے کا خطرہ)**

**57.1ـ کيا آپ کى ماں، والد، بھائى، بہن يا آپ کے اپنے بچوں ميں سے کسى کو ذيابيطس ہے؟**

*** ہاں  نہيں  اندازہ نہيں ہے***

اگر ہاں، تو کسے _____________________

**57.2ـ شرکا کا BMI ( BMI چارٹ کا استعمال )**

**57.3ـ کيا ڈاکٹر نے آپ کو کبھى بتايا کہ آپ کو ہائى بلڈ پريشر ہے يا اس کى ادويات ديں ہيں؟**

*** ہاں  نہيں  اندازہ نہيں ہے***

**57.4ـ قوميت؟ _______________________**

**57.5ـ ذيابيطس ہونے کے خطرے کا اعداد و شمار (ابتدائى ذيابيطس تشخيص کيلئے سکور) ؟ _______________**

(Finish Scoring Chart)

**58ـ مريض ذيابيطس ميں مبتلا ہے (سوال 6 سے)؟**

*** ہاں  نہيں _______ ہفتوں/مہينوں/سالوں***

***اگر ہاں؟ تو کيا آپ ذيابيطس کى ادويات کھارہے ہيں؟***

*** ہاں  نہيں اگر ہاں، تو کونسى ادويات___________________***

***اگر ذيابيطس نہيں ہے؟***

**RBG*سے سکرين کريں (گلوکوميٹر کے استعمال سے بلڈ ميں گلوکوز کا تجزيہ)***

**RBGکا نتيجہ ؟ ________________(mg/dl)**

اگر RBG 140-199 mg/dl سے زائد ہو تو OGTT کریں (**75 mg** ***گلوکوز کو پانی ملا کر*** مريض کو پلائیں اور دو گھنٹے کے بعد دوبارھ بلڈ میں ***گلوکوز کا تجزيہ کریں۔***

**PPBG کا نتيجہ ؟ ________________(mg/dl)**

اگر PPBG 140 mg/dl سے کم ہو تو یہ نارمل(Normal) ہے۔ اوراگر 140-199 mg/dl سے زائد ہو تو ***(pre-diabetic) پری ڈائیبیٹک*** *اور* 200 mg/dl سے زائد ہو تو علاج کيلئے مريض کو فزيشن / ذيابيطس ماہرکى طرف نتائج کے ساتھ بھيجيں۔

| *حصہ ـ* **K**  *علاج کا اختتام* |
| --- |

**59ـ علاج کا دورانيہ ختم ہونے کے بعد معيار زندگى:**

**59.1***۔ کيا آپ چلنے پھرنے کے قابل ہيں؟*

*** مجھے چلنے ميں كوئى مسئلہ نہيں ہے  مجھے چلنے ميں كچھـ مسئلہ ہے  ميں بستر تک محدود ہوں***

**59.2***۔ کيا آپ روزمرہ کام کرنے کے قابل ہيں؟ (جيسے کام، تعليم، گھريلو کام وغيرہ)*

*** مجھے روزمرہ کام کرنے ميں کوئى مشکل نہيں ہے  مجھے روزمرہ کام کرنے ميں تھوڑى مشکلات ہيں***

*** ميں روزمرہ کام کرنے کے قابل نہيں ہوں***

**59.3***۔ کيا آپ کو کسى درد / تکليف کا سامنا ہے ؟*

*** مجھے کوئى درد/تکليف نہيں ہے  مجھے تھوڑے درد/تکليف کا سامنا ہے  مجھے شديد درد/تکليف کا سامنا ہے***

**59.4***۔ کيا آپ اُداس / فکرمند ہيں؟*

*** ميں اُداس/فکرمند نہيں ہوں  ميں تھوڑا اُداس/فکرمند ہوں  ميں بہت اُداس/فکرمند ہوں***

**60ـ علاج کا دورانيہ ختم ہونے کے بعد کا ردعمل**

**60.1***۔ جن شکايات کے ساتھـ آيا ہے (نشانات اور علامات)؟*

*** ٹھيک ہوگئيں  کچھـ ٹھيک ہوئيں  ٹھيک نہيں ہوئيں***

**60.2***۔ علاج کا نتيجہ؟*

*** علاج مکمل ہوگيا  علاج کيلئے واپس نہيں آيا  علاج ناکام رہا  مريض کى وفات  شمارنہيں ہوا***
