# Supplementary material for: Predictors of slow clinical response and extended treatment in patients with extra-pulmonary tuberculosis in Pakistan, A hospital-based prospective study
Source: PLoS One. 2021 Nov 12;16(11):e0259801. doi: 10.1371/journal.pone.0259801 (PMC8589173; doi:10.1371/journal.pone.0259801)
Supplement: S3 File — (DOCX) [file pone.0259801.s003.docx]

**STUDY: IMPROVED DIAGNOSIS OF EXTRAPULMONARY TB**

**PATIENT REGISTRATION FORM**

**QUESTIONNAIRE- 0-14 years**

**(These questions to be asked from accompanying person (parents/relative/etc) with the child)**

**Date:**

**Paramedic/ Doctors** (who is interviewing the patient)**:**

**Hospital: Gulab Devi Hospital**

**Department:** OPD  IPD

**Extrapulmonary TB Suspect:**  Yes  No

| **INFORMED CONSENT** |
| --- |

**Informed consent (Part 1)**

** Yes  No**

***If Yes, complete sections A-F below.***

| **SECTION- A**  **PATIENT IDENTIFICATION** |
| --- |

**Name of patient:** ____________________________ **Study Number(three digit e.g.000)**: _______

**Age (years):**________

**Gender:** Male  Female

**Respondent:**  Parent  Other, relative/friend (write exact relationship) _____________________

**Address:** District __________City____________Village/Street/House____________________

| **SECTION- B**  **PERSONAL INFORMATION** |
| --- |

1. **Living with:**

one parent (mother/father) both parents  Relatives  Orphanage

Others, please specify____________________

1. **Level of education:**

 Not started formal education  started formal education but not completed primary school

 Completed primary school Completed middle school

 Completed secondary school Above secondary school

1. **Religion**:

Muslim  Christian  Other, please mention____________

1. **Has the child ever used chewable tobacco** (e.g.Pan, Niswar, Gutka)**:** Yes  No ­­­____weeks/months /years
2. **Has the child ever smoke cigarettes/ Huka (water pipe):** Yes  No ­­­____weeks/months /years

| **SECTION- C**  **PAST MEDICAL HISTORY** |
| --- |

1. **Does the child have any of these diseases?**

**Asthma:**  Yes  No

**Renal Disease:**  Yes  No

**Liver Diseases:**  Yes  No

**Diabetes Mellitus:**  Yes  No

**Hypertension:**  Yes  No

**Other:**  Yes  No

**Describe other: ­­­­­­­­­­­­­­**_____________________________

_____________________________

| **SECTION-D**  **HEALTH SEEKING BEHAVIOUR & DIAGNOSTIC DELAY** |
| --- |

***Health seeking behavior of TB patients***

***Please remind the attendant that this survey is confidential.***

1. **Please ask if the child has experienced any of the following symptoms**
   1. **General Symptoms**

**Fever:**  Yes  No ­­­____weeks/months

**What kind of fever does the child has?**  High-grade  Low-grade

**When do the child has fever?** Morning  Day-time  Evening  Night  all day

**Loss of weight:**   Yes  No ­­­____weeks/months

**Loss of appetite:**  Yes  No ­­­____weeks/months

**Night Sweat:**  Yes  No ­­­____weeks/months

**Fatigue:**  Yes  No ­­­____weeks/months

**Body weakness:**  Yes  No ­­­____weeks/months

**Frequent cold:**  Yes  No ­­­____weeks/months

**Neck mass:**  Yes  No ­­­____weeks/months

**Other:**  Yes  No ­­­____weeks/months

**Describe:_________________________**

- 1. **Respiratory Symptoms**

**Cough:**   Yes  No ___weeks/months

**Sputum:**  Yes  No ___weeks/months

**Cough with Sputum:**  Yes  No ___weeks/months

**Cough with blood:**  Yes  No ___weeks/months

**Chest pain:**  Yes  No ___weeks/months

**Difficulty in breathing:**  Yes  No ___weeks/months

- 1. **Abdominal Symptoms**

**Swelling of/in stomach:**  Yes  No ­­­ ___weeks/months

**Fullness of stomach:**  Yes  No ___weeks/months

**Vomiting:**  Yes  No ___weeks/months

**Diarrhea:**  Yes  No ___weeks/months

**Other:**  Yes  No ___weeks/months

**Describe other: ­­­­­­­­­­­­­­**____________________________________________

____________________________________________

- 1. **Neurological Symptoms**

**Headache:**  Yes  No ___weeks/months

**Photophobia:**  Yes  No ___weeks/months

**Vomiting:**  Yes  No ___weeks/months

**Dizziness:**  Yes  No ___weeks/months

**Vertigo:**  Yes  No ___weeks/months

**Weakness/Numbness of extremity:**  Yes  No ___weeks/months

**Visual disturbance:**  Yes  No ___weeks/months

**Other:**  Yes  No ___weeks/months

**Describe other: ­­­­­­­­­­­­­­**_____________________________

_____________________________

_____________________________

1. **What were the major symptoms in your child that first made you to seek care?**

 Prolong Cough  Coughing blood  Breathlessness

 Chest pain  Fever  Weight loss

 Fatigue\Weakness  Loss of appetite  Night sweats

Bone pain  Lymph node swelling Diarrhoea

Abdominal pain  others (specify) _________________

1. **When did you first notice the symptoms in child?**

______________________________________________________________________

1. **Did the child used any self-medication before you sought care?**  Yes  No
2. **When did you first time accompanied your child to seek medical advice for this illness after noticing the symptoms?**

**__________________________________ (days/weeks)**

1. **How many different places did you accompanied your child to seek help for the current symptoms? Number?______________and type of places?_________________**
2. **How many times have you accompanied your child to health facilities with the same symptoms before?**

 First visit  Second visit  Third visit  > 3 visits  don’t remember

1. **Which place did you first accompanied your child to seek care for his/her symptoms?**

 Tertiary Care Hospital  District hospital  Rural health center

 Private Hospital/clinic  Traditional healer  Pharmacy

 other, please specify___________________________________

1. **What kind of diagnosis did your child received for his/her illness**? _______________________
2. **Were any tests done at the first medical service?**

 Yes  No

1. **What type of tests?**

 Blood test  Urine test  Sputum  X-ray

Others, please specify_______________________________

1. **Did you take the results back from the doctor?**

 Yes  No

1. **Could you estimate the total cost for the previous visits/investigations related to your child’s current illness?**

Admission _________________________PKR

Consultations _________________________ PKR

Medication _________________________PKR

Laboratory tests/X-ray/CT________________________PKR

Transportation _________________________PKR

1. **Who has referred you to Gulab Devi Hospital?**

Self Traditional healers Religious leaders

Pharmacy/drug shop Village health worker Government dispensary

Government health center Government hospital Private dispensary/hospital

Charitable/NGO Member of the family Other___________________

1. **Before today, had you heard of the disease tuberculosis?**  Yes  No

**Pulmonary TB:**  Yes  No

**Extra pulmonary TB:**  Yes  No

1. **Does anyone in your family who has been diagnosed with TB before?**

 Yes  No

If yes? From where he/she has taken treatment? ____________________________

1. **Before today, had you heard of the illness tuberculosis?**

 Yes  No

1. **Do you know any symptoms of tuberculosis?**

 Chronic cough  Spitting blood  Shortness of breath

 Chest pain  Fever Weight loss

 Tiredness  Loss of appetite  Lymph nodes enlargement

 Others Please specify_____________________________***(Do not probe but askfor more symptoms)***

1. **Do you know which parts of the body that can be affected by tuberculosis?**

**____________________________________________________________________**

1. **Can tuberculosis spread from person to person?**

 Yes  No  Uncertain

1. **Has the child been in contact with a person with known tuberculosis?**

 Yes  No

If yes? Who was that person? ____________________________

1. **Does your child drink unboiled milk?**

 Yes  No

1. **Has your child received routine vaccination, by following the national children`s vaccination program**?

 Yes  No

1. **Has your child received BCG vaccination?**

 Yes  No

1. **Do people in your community stigmatize/ discriminate person having tuberculosis?**

 Yes  No  Uncertain

If yes, why? _________________________________

1. **Is there any thing that would make it easier for people with tuberculosis to get treatment,**

**not just in this clinic, but in other health facilities?**

 Yes  No  Uncertain

If yes, what could be done? ___________________________________________

1. **What fears do others have about TB that prevents them from seeking medical advice?**

__________________________________________________________________

| **SECTION- E**  **EXAMINATION** |
| --- |

1. **Physical signs**
   1. **General**

**Weight:** ______K.g.

**Temperature:** ______ Deg. Centigrade

**Pulse rate**: ______b.p.m

**Blood pressure:** __________

**Pallor:**  Yes  No

**Finger clubbing:**  Yes  No

**BCG scar:**  Yes  No

**Other:**  Yes  No

- 1. **Lymph nodes**

**Lymph node enlargement:**  Yes  No

**Matted:**  Yes  No

**Painful:**  Yes  No

**Discharge/Sinus:**  Yes  No

**Please draw enlarged lymph nodes or other findings:**


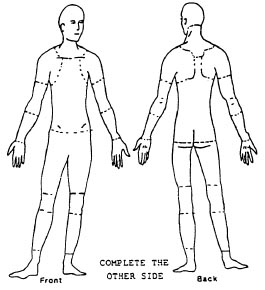


- 1. **Other Clinical Findings (as documented by the physicians/specialist)___________________**

**___________________________________________________________________________________**

| **SECTION- F**  **INVESTIGATIONS** |
| --- |

1. **Blood sample**

Hb ______________ ESR _____________

White blood cell count: ____________________________________________________________

1. **Bacteriology result (applicable if child can produce sputum)**
   1. **Sputum examination**

**AFB microscopy**

**Date(day.month.year) Appearance * Neg. + ++ +++**

**¯¯¯¯¯¯¯¯¯¯¯¯¯¯¯¯¯¯¯¯¯¯¯¯¯¯¯¯¯¯¯¯¯¯¯¯¯¯¯¯¯¯¯¯¯¯¯¯¯¯¯¯¯¯¯¯¯¯¯¯¯¯¯¯¯¯¯¯¯¯¯¯¯¯¯¯¯¯¯¯¯¯**

**Sample 1 (Spot-1)________________________________________________________________**

**Sample 2 (Morning)______________________________________________________________**

*visual appearance (blood stained, muo-purulent, saliva)

**MTB Culture**  Positive Negative

Date of positive culture (day, month, year):________________________

**GeneXpert**   Positive  Negative

RIF resistant Yes  No

- 1. **Other samples investigated**(pleura fluid, ascites, lymph node biopsies, FNA, CSF)

**Material: _____________________________________**

Laboratory serial number: __________________

**AFB microscopy:**   Positive Negative

**Cytology/histology: __________________________________________________________**

**___________________________________________________________________________**

**MTB Culture**:  Positive Negative

**MTP64:**  Positive Negative

**Biochemical tests**: Protein _________ Glucose_________ Cell count: _________________

**GeneXpert:**  Positive  Negative

RIF res  No  Yes

**Gram stain:________________________________ Bact. Culture:____________________________**

**Other tests:** _________________________________________________________________________

1. **Other Investigations**
   1. X-Ray Chest

________________________________________________________________________

________________________________________________________________________

- 1. Sonography/CT scan

________________________________________________________________________

________________________________________________________________________

- 1. Any other (specify): ___________________**______________________________**

| **SECTION-G**  **PATIENT WHO ARE REGISTERED FOR EPTB TREATMENT & FOLLOW-UP** |
| --- |

**TB Registration Number (TB03):** _________________

**Final Diagnosis: _**________________________________

**Patient Condition at the time of follow-up** (Clinically improved, Not improved, Somewhat improved):

| Follow-up 1. | Follow-up 3. | Follow-up 5. |
| --- | --- | --- |
| Follow-up 2. | Follow-up 4. | Follow-up 6. |

| **SECTION- H**  **QUALITY OF LIFE** |
| --- |

1. **Quality of Life (as time of registration):**
   1. **Does the child able to walk?**

 Has no problem in walking about Has some problem in walking about Confined to bed

- 1. **Does the child perform usual activity? (such as sports, studies, etc)**

 Has no problem with performing usual activity Has some problem with performing usual activity

 Unable to perform usual activity

- 1. **Does the child is having any pain/ discomfort?**

 Have no pain/discomfort Have moderate pain/discomfort  Have extreme pain/discomfort

- 1. **Does the child anxious/ depressed?**

 Not anxious/ depressed Moderately anxious/ depressed  Extremely anxious/ depressed

| **SECTION-I**  **CHILD GUARDIAN AND HOUSEHOLD COSTS** |
| --- |

***Estimate of the attendant/household income level***

1. **How long does it take you to go to the nearest health facility?**

 Less than 30 minutes between 30 minutes and one hour More than one hour

1. **How far is this hospital to your home (in Kilometers) _________________**
2. **How long (on average) does it take you to this health facility, waiting for**

**consultation and finally returning to home\workplace?** ________________Hours

1. **How did you get to this health facility?**

 Walked  Bicycle Motorcycle  Private car  Rikshaw/taxi  Bus

1. **If you have to take a public transport (e.g. Rikshaw /taxi/ bus)how much (on average) does it cost you to come to the clinic? ________________PKR.**
2. **Do you have to make some special arrangements at home before accompanying your child to the Hospital? For example: To look after your children back home in your absence, any disabled persons, pregnant women or any job related arrangements?**

 Yes  No  Uncertain

If yes, what arrangements?­­­­­­­­­­­­­­­­­__________________________

1. **What is the main occupation of this child’s guardian? (past twelve months)?**

 Employed by government  Employed private

 Self-employed (mention the self-employment such as: merchant/shop keeper/ farmer/ fishing/property agent/ etc)___________  Student  Housewife  Other_________________

1. **What is the main source of income of this child’s guardian and their house holds?**

 Employment (Govt or private)  Pensions

 Crop production  Livestock  Fishing

 Hunting/ bee-keeping  Poultry  Farm wage

 Other agricultural activity  Wages (government)  Wages (private)

 Monetary savings (interest)  Property (rentals) Self-employed payments (merchant)Other Specify____________________________

1. **How much did the guardian of this child (NAME) earn (money) for the activities stated on average in the past 12 months? This should include not only salary or cash income: but also the value of goods produced or traded for other goods and services.**

**_____________________________________________________________________**

Between PKR:

Less than 10,000

 10,000 – 20,000

 21,000-30,000

 31,000 – 40,000

 41,000-50,000

 More than 50,000

1. **Does guardian of this child own a house?**

 Yes renting a house  living with relatives /friends  Homeless

1. **How many people live in that household:** _________________ (number of people)
2. **What is the main source of drinking water for members of this child’s household?**

 Piped water 1=Piped into dwelling 2= Piped into yard/plot 3=Public tap 4=Neighbors’ tap

 Hand Pump

 Water supplied by Tanker/Truck

 Water from open well

 Tube well/Turbine

 Running water 1=spring; 2=river/stream; 3=pond/Lake; 4=Dam

 Rain water

Water vendor

Bottled water

 Others Specify ________________________

1. **What kind of toilet facilities do members of your household usually use?**

Flush to piped sewer systemFlush to septic tank

 Open Pit  Ventilated improved pit (VIP)  Public Latrine

 No facility/bush/field  other, please specify ______________

1. **Does your household have?**

 Electricity  Gas  Radio  Television  Telephone/mobile  Iron (either charcoal or electricity)  Refrigerator

1. **What is the main source of energy for lighting in your household?**

 Main electricity  Solar  Gas  Kerosene lamp

 Firewood  Candles  other, please specify _________________________

1. **What is the main material for the walls of your house or house you are living?**

 Mud  Cement bricks  Backed bricks  Wood

Stones  Others Specify_________________________

1. **What is the roofing material of your house or house you are living?**

 Grass/leaves/mud  Iron sheets  Tiles  Concrete/Cement

 Others Specify_______________

1. **Does any member of your household own?**

 A bicycle  A motorcycle or motor scooter  A car  A bank account

1. **How many acres of land for farming/grazing are owned by the household?**

 Arable land_____________acres Land for grazing__________acres

1. **How many meals does your household usually have per day?**

Meals (in number)?_________________

**Informed consent (Part 2- for Blood Dry Spot)**

** Yes  No**

***If Yes, take the blood sample on paper and store as per the guidelines.***

| **SECTION- J**  **DIABETES SCREENING** |
| --- |

1. **Pre-diabetic (risk of getting diabetes)**
   1. **Does any of the family members of the child (mother or father or brother or sister) has diabetes?**

 Yes  No  Uncertain

If yes, who ____________________

- 1. **BMI of child [Use the BMI chart]? ___________ _____**
  2. **Has a doctor ever told that the child has high blood pressure, or given medication for it?**

 Yes  No  Uncertain

- 1. **Nationality? ______________________**
  2. **Risk calculation (score to establish as pre-diabetic)?__________________**

**(Use Finnish Scoring chart)**

1. **Child is known diabetic (from question 6)?**

 Yes  No ______ weeks/months/ years

If yes? Is he/she taking medication for diabetes?

 Yes  No If yes? Which medicines?______________

**If unknown diabetic?**

**Screen with**

**Random Blood Glucose (using gluco-meter)**

**Result of RBG? ___________ (mg/dl)**

**If RBG** $\boldsymbol{\geq}$ **140-199 mg/dl , perform OGTT (offer 75mg of glucose dissolved in water and check blood sugar after 2 hours)**

**Result of PPBG? ___________ (mg/dl)**

**Screen also with FBG, by asking patient/attendant to come to the hospital next morning with empty *If PPBG <140 mg/dl (normal), if*** $\boldsymbol{\geq}$ **140-199 *mg/dl (pre-diabetic) and if*** $\boldsymbol{\geq}$ **200 mg/dl *refer the patient to physician/ Diabetes Specialist with patient results***

| **SECTION- K**  **END OF TREATMENT** |
| --- |

1. **Quality of Life (at end of treatment duration):**
   1. **Does the child able to walk?**

 Have no problem in walking about Have some problem in walking about Confined to bed

- 1. **Does the child able to perform usual activity? (such as studies, sports, etc)**

 Has no problem with performing usual activity Has some problem with performing usual activity

 Unable to perform usual activity

- 1. **Does the child have any pain/ discomfort?**

 Have no pain/discomfort Have moderate pain/discomfort  Have extreme pain/discomfort

- 1. **Does the child anxious/ depressed?**

 Not anxious/ depressed Moderately anxious/ depressed  Extremely anxious/ depressed

1. **Response to treatment (at end of treatment duration):**
   1. **Presenting complaints (signs and symptoms)?**

 Settled  Somewhat settled  Not settled

- 1. **Treatment outcome?**

 Treatment completed  Lost to follow-up  Treatment Failure  Died  Not Evaluated
