# Supplementary material for: Predictors of slow clinical response and extended treatment in patients with extra-pulmonary tuberculosis in Pakistan, A hospital-based prospective study
Source: PLoS One. 2021 Nov 12;16(11):e0259801. doi: 10.1371/journal.pone.0259801 (PMC8589173; doi:10.1371/journal.pone.0259801)
Supplement: S4 File — (DOCX) [file pone.0259801.s004.docx]

تحقيق : بيرون پھيپھڑوں والى ٹى بى کى تشخيص ميں بہترى

*مريض کا رجسٹريشن فارم*

*سوالنامه:* **0** *سے 14 سال كے لوگوں كيلئے*

*يہ سوالات بچے کے ساتھـ آنے والے فرد (والدين/رشتہ دار وغيرہ) سے پوچھيں جائيں۔*

*تاريخ:*

*پيراميڈک / ڈاكٹر (جس نے مريض سے انٹرويو ليا)*

*هسپتال: گلاب ديوى ہسپتال*

*شعبہ:  او پى ڈى  آئى پى ڈى*

*بيرون پھيپھڑوں والى ٹى بى کى تشخيص  ہاں  نہيں*

| *سوالنامہ / انٹرويو کے ليے رضامندى* |
| --- |

***كيا آپ اس سوالنامه/انٹرويو كيلئے رضامند ہيں (*حصه *اول)***

*** ہاں  نہيں***

***اگر جواب ہاں ميں ہو تو درج ذيل* A-F *حصوں كو مكمل كريں۔***

| **حصه ـ A**  مريض كى شناخت |
| --- |

***مريض كا نام : ____________________________ سٹڈى نمبر (تين ہندسے مثلا 000)___________________***

***عمر* *(سال)* ______________**

***جنس  لڑكا  لڑكى***

***جواب دينے والا  والدين  ديگر، رشتہ دار/دوست (اصل رشتہ لکھيں) _________________***

***پتہ: ضلع ________________ شہر _____________ گاؤں/گلى/گھر_________________________***

| **حصه ـB**  ***ذاتى معلومات*** |
| --- |

*1۔ کس کے ساتھـ رہائش پذير ہے؟*

*** کسى ايک کے ساتھـ (والد/والدہ)  والدين کے ساتھـ  رشتہ دار *** *يتيم خانہ*

*** ديگر، وضاحت كريں __________________***

*2۔ تعليمى معيار*

*** رسمى تعليم نہيں لى  پرائمرى سكول مكمل نہيں كيا***

*** پرائمرى پاس  مڈل پاس***

*** ميٹرک پاس  ميٹرک سے اوپر***

*** تعليم بالغاں  ديگر (درج كريں)__________________________***

*3۔ مذہب*

*** مسلمان  عيسائى  ديگر كوئى، (درج كريں) ______________________***

*4۔ كيا بچے نے کبھى چبانے والا تمباكو استعمال کيا ہے (مثلا پان، نسوار، گٹكا)*

*** ہاں  نہيں _____ہفتوں /مہينوں / سالوں سے***

*5۔ كيا بچے نے کبھى سگريٹ / حقہ پيا ہے؟*

*** ہاں  نہيں _____ہفتوں /مہينوں / سالوں سے***

| حصہ ـ **C**  *ماضى کے طبى مسائل* |
| --- |

**6*۔ کيا بچے کو درج ذيل ميں سے کوئى بيمارى ہے؟***

*دمہ* *** ہاں  نہيں***

*گردوں کى بيمارى****:  ہاں  نہيں***

*ذيابيطس (شگر) کى بيمارى****:  ہاں  نہيں***

***بلڈ پريشر/ بلند فشار خون  ہاں  نہيں***

***ديگر  ہاں  نہيں***

***وضاحت کريں ___________________________***

***___________________________***

| *حصہ ـ* **D**  *علاج کے حصول کا رويہ اور تشخيص ميں تاخير* |
| --- |

**ٹى بى کے مريض کا علاج کے حصول کا رويہ**

**مريض کے ساتھـ آنے والے کو بتائيں کہ اس سوالنامہ / انٹرويو کو پوشيدہ رکھا جائے گا۔**

**7ـ پوچھيں کہ کيا بچے نے درج ذيل علامات کا سامنا کيا**

**7.1 عام علامات**

بخار: *** ہاں  نہيں _______ ہفتے / مہينے***

***بچے کو کيسا بخار چڑھتا ہے:  زيادہ شدت کا  کم شدت کا***

***بچے کو کس وقت بخار چڑھتا ہے:  صبح  دن  شام  رات  تمام دن***

***وزن ميں کمى  ہاں  نہيں _______ ہفتے / مہينے***

***بھوک نہ لگنا  ہاں  نہيں _______ ہفتے / مہينے***

***رات کو پسينہ  ہاں  نہيں _______ ہفتے / مہينے***

***تھکاوٹ  ہاں  نہيں _______ ہفتے / مہينے***

***حيض بند ہونا (صرف لڑکى کيلئے)  ہاں  نہيں _______ ہفتے / مہينے***

***جسمانى کمزورى  ہاں  نہيں _______ ہفتے / مہينے***

***سردى کا زيادہ لگنا  ہاں  نہيں _______ ہفتے / مہينے***

***گردن ميں غدود  ہاں  نہيں _______ ہفتے / مہينے***

***ديگر کوئى  ہاں  نہيں _______ ہفتے / مہينے***

***وضاحت کريں ______________________________***

**7.2 سانس (نظام تنفس) کى علامات**

***کھانسى  ہاں  نہيں _______ ہفتے / مہينے***

***بلغم  ہاں  نہيں _______ ہفتے / مہينے***

***کھانسى کے ساتھـ بلغم  ہاں  نہيں _______ ہفتے / مہينے***

***بلغم کے ساتھـ خون  ہاں  نہيں _______ ہفتے / مہينے***

***سينے ميں درد  ہاں  نہيں _______ ہفتے / مہينے***

***سانس لينے ميں تکليف  ہاں  نہيں _______ ہفتے / مہينے***

**7.3 پيٹ کى علامات**

***معدے پر/ميں سوجن  ہاں  نہيں _______ ہفتے / مہينے***

***معدہ بھرا ہونا  ہاں  نہيں _______ ہفتے / مہينے***

***قے/اُلٹى  ہاں  نہيں _______ ہفتے / مہينے***

***اسھال  ہاں  نہيں _______ ہفتے / مہينے***

***ديگر  ہاں  نہيں _______ ہفتے / مہينے***

***وضاحت کريں ________________________________***

**7.4 اعصابى علامات**

***سر درد  ہاں  نہيں _______ ہفتے / مہينے***

***روشنى سے ڈر  ہاں  نہيں _______ ہفتے / مہينے***

***قے/اُلٹى  ہاں  نہيں _______ ہفتے / مہينے***

***چکر  ہاں  نہيں _______ ہفتے / مہينے***

***نقاہت  ہاں  نہيں _______ ہفتے / مہينے***

***کمزورى/ سن ہونا  ہاں  نہيں _______ ہفتے / مہينے***

***نظر خراب ہونا  ہاں  نہيں _______ ہفتے / مہينے***

***ديگر  ہاں  نہيں _______ ہفتے / مہينے***

***وضاحت کريں ______________________________***

***______________________________***

***______________________________***

**8ـ وہ کونسى اہم علامات تھيں جن کى وجہ سے آپ اپنے بچے کے علاج کى طرف متوجہ ہوئے**

*** طويل کھانسى  بلغم کے ساتھـ خون  سانس چڑھنا***

*** سينے ميں درد  بخار  وزن ميں کمى***

*** تھکاوٹ/کمزورى  بھوک نہ لگنا  رات کو پسينہ***

*** ہڈيوں ميں درد  لمف نوڈ سوجن  ڈائريا / اسھال***

*** پيٹ کا درد  ديگر (وضاحت کريں) ______________________***

**9ـ آپ نے پہلى مرتبہ اپنے بچے ميں کب يہ علامات محسوس کيں؟**

***_____________________________________________________________________________***

**01ـ کيا بچے نے علاج سے قبل اپنے طور پر ادويات استعمال کيں۔ * ہاں  نہيں***

**11ـ بيمارى کى علامات محسوس کرنے کے کتنے عرصے بعد آپ نےاپنے بچے کيلئے طبى مشورہ ليا؟**

***________________________ دن /***  *ہفتے*

**12ـ اپنے بچے کى موجودہ علامات کا علاج کرانے کيلئے آپ کتنى جگہوں پر مدد کيلئے گئے؟**

***تعداد؟ _________________ اور جگہوں کى نوعيت؟_________________________***

**13ـ اس سے قبل اپنے بچے علاج کيلئے انھى علامات کے ساتھـ کتنى مرتبہ مراکز صحت کا چکر لگايا ؟**

*** پہلا چکر  دوسرا چکر  تيسرا چکر***

*** تين سے زائد چکر  ياد نہيں***

**14ـ اپنے بچے کى علامات کے علاج کيلئے آپ اُسے پہلى مرتبہ کہاں لے کر گئے؟**

*** درجہ سوم نگہداشتى ہسپتال (بڑا ہسپتال)  ضلعى ہسپتال  ديہى مرکز صحت***

*** نجى ہسپتال/کلينک  روايتى حکيم  فارميسى***

*** ديگر، وضاحت کريں _______________________________________***

**15ـ بچے کى تکليف/علامات سے کونسى بيمارىتشخيص ہوئى؟** __________________________________

**16ـ پہلى طبى ملاقات پر آپ کے کوئى ٹيسٹ کرائے گئے ؟**

*** ہاں  نہيں***

**17ـ کس نوعيت کے ٹيسٹ ہوئے؟**

*** خون کا ٹيسٹ  پيشاب کا ٹيسٹ  بلغم/تھوک کا ٹيسٹ  ايکسرے***

*** ديگر، وضاحت کريں _______________________________________***

**18ـ کيا آپ ڈاکٹر کے پاس ٹيسٹوں کى رپورٹ لے کر گئے؟**

*** ہاں  نہيں***

**19ـ کيا آپ اپنى موجودہ بيمارى سے متعلق گزشتہ چکر / تشخيص کے اخراجات کا اندازہ لگاسکتے ہيں؟**

***داخلہ ___________________ روپے***

***مشاورتى فيس ___________________ روپے***

***ادويات ___________________ روپے***

***ليبارٹرى ٹيسٹ/ايکسرے/سى ٹى ___________________ روپے***

***سفرى اخراجات ___________________ روپے***

**20ـ گلاب ديوى ہسپتال ميں علاج کيلئے کس نے بھيجا؟**

*** خود  روايتى حکيم  مذہبى رہنما***

*** فارميسى/ادويات کى دکان  ديہى ہيلتھـ ورکر  سرکارى ڈسپنسرى***

*** سرکارى مرکز صحت  سرکارى ہسپتال  نجى ڈسپنسرى/ہسپتال***

*** خيراتى مرکز/اين جى او  خاندان کے فرد  ديگر__________________***

**21ـ آج سے قبل کيا آپ نے تپ دق (ٹى بى) کے بارے ميں سنا تھا؟** * ہاں  نہيں*

***پھيپھڑوں والى ٹى بى  ہاں  نہيں***

***بيرون پھيپھڑوں والى ٹى بى  ہاں  نہيں***

**22ـ کيا اس سے قبل آپ کے خاندان ميں کسى کو ٹى بى کا مرض ہوا؟**

*** ہاں  نہيں***

***اگر ہاں؟ تو اس نے کہاں سے علاج کروايا؟ _______________________________________***

**23ـ کيا آپ تپ دق (ٹى بى) کی کسی علامات کے بارے میں جانتے ہیں؟**

*** طويل کھانسى  بلغم کے ساتھـ خون  سانس چڑھنا***

*** سينے ميں درد  بخار  وزن ميں کمى***

*** تھکاوٹ/کمزورى  بھوک نہ لگنا  لمف نوڈ سوجن***

*** ديگر (وضاحت کريں) ______________________***

***(تحقیق کی بجائے مزید علامات جاننے کے لئے مزید سوالات کریں؟)***

**24ـ کيا آپ جانتے ہیں کہ تپ دق (ٹى بى) کی وجہ سے آپ کے جسم کے کونسے حصے متاثرہو سکتے ہیں؟**

***____________________________________________________________________***

**25ـ کیا تپ دق (ٹى بى) ایک شخص سے دوسرے شخص کو لگ سکتی ہے؟**

*** ہاں  نہيں***

**26ـ کيا آپ دودھ کو اُبالے بغير پيتے ہيں؟**

*** ہاں  نہيں***

**27ـ کيا آپ کا بچہ ایسے شخص کے رابطے میں ہے جس کو تپ دق (ٹى بى) ہے؟**

*** ہاں  نہيں***

***اگر ہاں تو وہ کون شخص ہے؟***

***_____________________________________________***

**28ـ کيا آپ کا بچہ بغير اُبلا دودھ پيتا ہے؟**

*** ہاں  نہيں***

**92ـ کيا آپ کے بچے کو نيشنل چلڈرن ويکسينيشن پروگرام کے تحت باقاعدگى سے ويکسين دى گئى ہے؟**

*** ہاں  نہيں***

**30ـ کيا آپ کے بچےکو BCG ويکسين دى گئى ہے؟**

*** ہاں  نہيں***

**31ـ کيا آپ کے خاندان/علاقہ ميں لوگ تپ دق (ٹى بى) کے مريض سے اچھا برتاؤ نہيں رکھتے؟**

*** ہاں  نہيں  اندازہ نہيں ہے***

***اگر ہاں؟ تو کيوں؟ _________________________________________***

**32ـ اس کلينک کے ساتھـ ساتھـ ديگر مراکز صحت ميں ايسا کيا کيا جائے جس سے تپ دق (ٹى بى) کے مريضوں کو علاج ميں آسانى ہو؟**

*** ہاں  نہيں  اندازہ نہيں ہے***

***اگر ہاں؟ تو کيا ہوسکتا ہے؟ ___________________________________________________***

**33ـ ٹى بى کے بارے ميں دوسرے لوگوں کو کس قسم کے خدشات لاحق ہيں جو اُنھيں طبى مشورے سے دور رکھتے ہيں ؟**

***_______________________________________________________________________***

| *حصہ ـ* **E**  *معائنہ* |
| --- |

**34ـ جسمانى علامات**

**34.1 عام علامات**

وزن _______ کلوگرام

حرارت _______ ڈگرى سينٹى گريڈ

نبض کى رفتار _______ دھڑکن فى منٹ

بلڈپريشر _______

رنگت *** ہاں  نہيں***

انگليوں کا ارتعاش *** ہاں  نہيں***

BCG نشان *** ہاں  نہيں***

ديگر *** ہاں  نہيں***

**34.2 موادي گلٹياں (لمف نوڈز)**

لمف نوڈز /موادى گلٹياں بڑى ہونا *** ہاں  نہيں***

الجھا ہوا *** ہاں  نہيں***

تکليف *** ہاں  نہيں***

سوراخ/ اخراج والا *** ہاں  نہيں***

***برائے مہربانى موادى گلٹيوں (لمف نوڈز) يا ديگر نتائج کى تصوير ميں نشاندہى کريں۔***


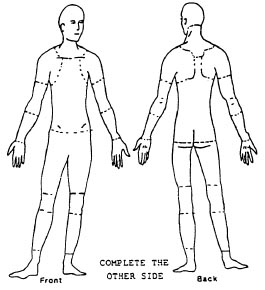


**34.3 ديگر طبى نتائج (جو ڈاکٹر/ماہر نے درج کيے ہوں)**____________________________________

________________________________________________________________________

| *حصہ ـ* **F**  *تحقيقات کے نتائج* |
| --- |

**35ـ خون کا نمونہ**

Hb__________________ ESR ________________

خون ميں سفيد خليوں کى تعداد _________________________________________________________

**36ـ بيکٹيريالوجى نتيجہ (اگر بچہ تھوک/بلغم نمونے کے طور پر دے سکتا ہے)**

**36.1 بلغم کا تجزيہ**

AFBمائيکروسکوپى

تاريخ (سال ۔ ماہ ـ دن) شکل***** منفى۔ **+ ++ +++**

_____________________________________________________________________________

نمونہ 1: (سپاٹ ـ 1) _______________________________________________________________

نمونہ 2: (صبح) _________________________________________________________________

***** کس شکل ميں ہے (خون آلود، پيپ، تھوک)

**MTB کلچر**  *** مثبت  منفى***

***مثبت کلچر کى تاريخ (سال، ماہ، دن) ______________________***

**GeneXpert * مثبت  منفى***

RIF مزاحمت *** ہاں  نہيں***

**36.2 ديگر نمونے (پلورا فلوڈ، ايسيٹيس، لمف نوڈ بائيوسپيس، FNA، CSF)**

مواد: ___________________

ليبارٹرى سيريل: ___________

AFBمائيکروسکوپى *** مثبت  منفى***

***سائيٹالوجى/ہسٹالوجى _________________________________________________________***

***_______________________________________________________________________***

MTBکلچر: *** مثبت  منفى***

MTP64: *** مثبت  منفى***

بائيوکيميکل ٹيسٹ: پروٹين_____________ گلوکوز___________ خليون/ سيل کى تعداد ____________

GeneXpert: *** مثبت  منفى***

RIF res *** نہيں  ہاں***

***گرام سٹين ________________________________ بيکٹريل کلچر __________________________***

***ديگر ٹيسٹ ___________________________________________________________________***

**37ـ ديگر ٹيسٹ**

**37.1 ايکسرے چھاتى/سينہ**

***__________________________________________________________________________***

***__________________________________________________________________________***

**37.2 سونوگرافى / سى ٹى سکين**

***__________________________________________________________________________***

**37.3 ديگر، وضاحت کريں،*______________________________________________________***

***__________________________________________________________________________***

| *حصہ ـ* **G**  *بيرون پھيپھڑوں والى ٹى بى کے علاج کیلیے مریض کی رجسٹریشن اور اس کا مرحلہ وار علاج* |
| --- |

**:(TB03)** *ٹی بی رجسٹریشن نمبر*

*مکمل تشخیص:*

*کے وقت مریض کی حالت :* **(Follow-up)***علاج مرحلہ وار*

*( طبی لحاظ سے بہتری آئی ہے،بلکل بہتری نہیں آئی، کچھ حد تک بہتری آئی ہے)*

| Follow-up 1. | Follow-up 3. | Follow-up 5. |
| --- | --- | --- |
| Follow-up 2. | Follow-up 4. | Follow-up 6. |

| *حصہ ـ* **H**  *مريض کی حالت* |
| --- |

**38***۔ رجسٹريشن كے وقت مريض کى حالت*

**38.1***۔ کيا بچہ چلنے پھرنے کے قابل ہے؟*

*** چلنے ميں كوئى مسئله نہيں ہے  چلنے ميں كچھـ مسئلہ ہے  بستر تک محدود ہے***

**38.2***۔ کيا بچہ روزمرہ معاملات کے قابل ہے؟ (جيسے کھيل، تعليم وغيرہ)*

*** روزمرہ معاملات ميں کوئى مشکل نہيں ہے  روزمرہ معاملات ميں تھوڑى مشکلات ہيں***

*** روزمرہ معاملات کے قابل نہيں ہے۔***

**38.3***۔ کيا بچہ و کسى درد / تکليف کا سامنا ہے ؟*

*** کوئى درد/تکليف نہيں ہے  تھوڑے درد/تکليف کا سامنا ہے  شديد درد/تکليف کا سامنا ہے***

**38.4***۔ کيا بچہ اُداس / فکرمند ہے؟*

*** اُداس/فکرمند نہيں ہے  تھوڑا اُداس/فکرمند ہے  بہت اُداس/فکرمند ہے***

| *حصہ ـ* **I**  *بچے کا سرپرست اور گھريلو اخراجات* |
| --- |

*ساتھـ آنے والے فرد / گھرانے کى آمدن کا اندازہ*

**39۔** قريبى مرکز صحت جانے ميں آپ کا کتنا وقت لگتا ہے؟

*** 30 منٹ سے کم  آدھے سے ايک گھنٹہ  ايک گھنٹے سے زائد***

**40**۔ آپ کے گھر سے ہسپتال کا فاصلہ (کلوميٹرميں) کتنا ہے؟ **_______________________**

**41**۔ مرکز صحت آنے، مشاورت کيلئے اپنى بارى کے انتظار اور واپس گھر/کام پر پہنچنے ميں عموماً کتنا

وقت لگتا ہے؟ **____________ گھنٹے**

**42**۔ آپ مرکز صحت کس طرح آتے ہيں؟

*** پيدل  سائيکل  موٹرسائيکل  ذاتى گاڑى  رکشہ/ٹيکسى  بس***

**43**۔ کلينک آنے کيلئے پبلک ٹرانسپورٹ (مثلاً رکشہ/ٹيکسى/بس) استعمال کريں تو عموماً کتنى رقم

خرچ ہوتى ہے؟ **____________ روپے**

**44**۔ کيا بچے کے ساتھـ ہسپتال آنے سے پہلے آپ کو گھر ميں کچھـ خصوصى انتظامات کرنے پڑتے ہيں؟ مثلاً آپ کى غيرموجودگى ميں بچوں، کسى معذور فرد يا حاملہ عورت کى ديکھـ بھال يا نوکرى سے متعلق کوئى انتظامات؟

*** ہاں  نہيں  اندازہ نہيں ہے***

***اگر ہاں؟ تو کيسے انتظامات؟ ___________________________________________________***

**45**۔ گزشتہ ايک سال سے بچے کے سرپرست کا اصل پيشہ کيا ہے؟

*** سرکارى ملازم  پرائيويٹ ملازم***

*** اپنا کاروبار (اپنے کاروبار جيسے مرچنٹ/دکاندار/کسان/ماہى گيرى/پراپرٹى ايجنٹ وغيرہ درج کريں)_________***

*** طالبعلم  خاتون خانہ  ديگر ________________***

**46ـ بچے کے سرپرست / گھرانے کا بنيادى ذريعہ آمدن کيا ہے؟**

*** ملازمت (سرکارى/پرائيويٹ)  پينشن***

*** فصلوں کى کاشت  مويشى پالنا  ماہى گيرى***

*** شکارکرنا  پولٹرى فارم  فارم پر اجرتى ملازم***

*** ديگر زرعى سرگرمياں  اجرت (سرکارى)  ديہاڑى (پرائيويٹ)***

*** مالياتى بچت (سود)  جائيداد کا کرايہ  اپنا کاروبار (دکاندار)***

*** ديگر__________________***

**47**۔ گزشتہ ايک سال ميں ان کاموں سے بچے کے سرپرست کى اوسط ماہانہ آمدن کيا ہے؟اس ميں صرف تنخواہ يا نقد آمدن ہى نہيں بلکہ پيداوار يا تجارتى اشياء کى قيمت اور خدمات بھى شامل کى جائيں۔

_____________________________________________________________________________

تقريباً پاکستانى روپے

*** 10000 سے کم***

*** 10000 سے 20000***

*** 21000 سے 30000***

*** 31000 سے 40000***

*** 41000 سے 50000***

*** تقريباً 50000 يا اس سے زيادہ***

**48۔** کيا بچے کے سرپرست کا رہائشى گھر اپنا ہے؟

*** ہاں  کرائے کا گھر  رشتہ دار / دوست کے ساتھـ رہائش  بے گھر***

**49**۔ اس گھر ميں کتنے افراد رہائش پذير ہيں؟ ________________ (گھر کے افراد)

**50**۔ بچے کے گھر ميں پينے والے پانى کا بنيادى ذريعہ کيا ہے؟

*** پائپ لائين 1= گھر ميں پائپ لائين 2= صحن/پلاٹ ميں پائپ لائين 3= سرکارى نلکا 4= پڑوسيوں کا نلکا***

*** ہينڈ پمپ***

*** ٹينکر/ٹرک سے پانى کى فراہمى***

*** کھلے کنويں کا پانى***

*** ٹيوب ويل / ٹربائين***

*** چلتا پانى 1= چشمہ 2= دريا/ندى 3= تالاب/جھيل 4= ڈيم***

*** بارشى پانى***

*** پانى بيچنے والا***

*** بوتلوں کا پانى***

*** ديگر، وضاحت کريں ________________________________***

**51ـ عام طور پر آپ کے گھر والے کس قسم کا بيت الخلا/باتھـ روم استعمال کرتے ہيں؟**

*** پائپوں والا سيوريج سسٹم  سيپٹک ٹينک ميں نکاس***

*** کھلا گڑھا  ہوادار بہترين گڑھا***(VIP) *** عوامى بيت الخلا/ ليٹرين***

*** سہولت نہيں ہے/جھاڑياں/کھيت  ديگر، وضاحت کريں____________________***

**52ـ کيا آپ کے گھر ميں درج ذيل اشياء ہيں؟**

*** بجلى  گيس  ريڈيو  ٹيلى ويژن  فون/ موبائيل***

*** استرى (چاہے کوئلے يا بجلى والى)***

**53ـ آپ کے گھر ميں روشنى کيلئے توانائى کا بنيادى ذريعہ کيا ہے؟**

*** بجلى  شمسى توانائى  گيس  مٹى کے تيل والا ليمپ***

*** لکڑيوں کى آگ  موم بتى  ديگر، وضاحت کريں ______________________***

**54ـ آپ کے گھر يا رہائشى جگہ کى ديواريں کس چيز سے بنى ہوئى ہيں؟**

*** کيچڑ/گارا  سيمنٹ کى اينٹيں  پکى اينٹيں  لکڑى***

*** پتھر  ديگر، وضاحت کريں ______________________***

**55ـ آپ کے گھر يا رہائشى جگہ کى چھتيں کس چيز سے بنى ہوئى ہيں؟**

*** گھاس/پتے/کيچڑ  جستى چادريں  ٹائليں  کنکريٹ/سيمنٹ***

*** ديگر، وضاحت کريں ______________________***

**56ـ کيا آپ يا گھر کے کسى فرد کے پاس درج ذيل اشياء موجود ہيں؟**

*** سائيکل  موٹرسائيکل/سکوٹر  کار  بنک اکاؤنٹ***

*** ديگر، وضاحت کريں ______________________***

**57ـ آپ کے گھرانے کے پاس کاشتکارى/گلہ بنانى کيلئے کتنے ايکڑ زمين موجود ہے؟**

*** قابل کاشت زمين ________ ايکڑ  چرانے کيلئے زمين ________ ايکڑ***

**58ـ آپ کے گھروالے عام طور پر روزانہ کتنى مرتبہ کھانا کھاتے ہيں؟**

*** کھانوں کى تعداد (نمبروں ميں) _____________________***

*كيا آپ رضامند ہيں (*حصه *دوم) خشک خون کا نمونہ*

*** ہاں  نہيں***

| *حصہ ـ* **H**  *ذيابيطس سکريننگ* |
| --- |

**59ـ ابتدائى ذيابيطس (زيابيطس ہونے کا خطرہ)**

**59.1ـ کيا بچے کى والدہ، والد، بھائى يا بہن ميں سے کسى کو ذيابيطس ہے؟**

*** ہاں  نہيں  اندازہ نہيں ہے***

اگر ہاں، تو کسے _____________________

**59.2ـ بچے کا BMI ( BMI چارٹ کا استعمال )**

**59.3ـ کيا ڈاکٹر نے کبھى بتايا کہ بچے کو ہائى بلڈ پريشر ہے يا اس کى ادويات ديں ہيں؟**

*** ہاں  نہيں  اندازہ نہيں ہے***

**59.4ـ قوميت؟ _______________________**

**59.5ـ ذيابيطس ہونے کے خطرے کا اعداد و شمار (ابتدائى ذيابيطس تشخيص کيلئے سکور) ؟ _______________**

**60ـ کيا بچہ ذيابيطس ميں مبتلا ہے (سوال 6 سے)؟**

*** ہاں  نہيں _______ ہفتوں/مہينوں/سالوں***

***اگر ہاں؟ تو کيا آپ ذيابيطس کى ادويات کھارہے ہيں؟***

*** ہاں  نہيں اگر ہاں، تو کونسى ادويات___________________***

***اگر ذيابيطس نہيں ہے؟***

**RBG*سے سکرين کريں (گلوکوميٹر کے استعمال سے بلڈ ميں گلوکوز کا تجزيہ)***

**RBGکا نتيجہ ؟ ________________(mg/dl)**

اگر RBG 140-199 mg/dl سے زائد ہو تو OGTT کریں (**75 mg** ***گلوکوز کو پانی ملا کر*** مريض کو پلائیں اور دو گھنٹے کے بعد دوبارھ بلڈ میں ***گلوکوز کا تجزيہ کریں۔***

مريض کو اگلے دن صبح خالى پيٹ آنے کا کہہ کر FBG ***(گلوکوميٹر کے استعمال سے فاسٹنگ بلڈ ميں گلوکوز کا تجزيہ)*** سے بھى سکرين کريں۔

**PPBG کا نتيجہ ؟ ________________(mg/dl)**

اگر PPBG 140 mg/dl سے کم ہو تو یہ نارمل(Normal) ہے۔ اوراگر 140-199 mg/dl سے زائد ہو تو ***(pre-diabetic) پری ڈائیبیٹک*** *اور* 200 mg/dl سے زائد ہو تو علاج کيلئے مريض کو فزيشن / ذيابيطس ماہرکى طرف نتائج کے ساتھ بھيجيں۔

| *حصہ ـ* **K**  *علاج کا اختتام* |
| --- |

**61ـ علاج کا دورانيہ ختم ہونے کے بعد معيار زندگى:**

**61.1***۔ کيا بچہ چلنے پھرنے کے قابل ہے؟*

*** چلنے ميں كوئى مسئلہ نہيں ہے  چلنے ميں كچھـ مسئلہ ہے  بستر تک محدود ہے***

**61.2***۔ کيا بچہ روزمرہ معاملات کے قابل جيسے؟ (جيسے کھيل، تعليم وغيرہ)*

*** روزمرہ معاملات ميں کوئى مشکل نہيں ہے  روزمرہ معاملات ميں تھوڑى مشکلات ہيں***

*** روزمرہ معاملات کے قابل نہيں ہے***

**61.3***۔ کيا بچے کو کسى درد / تکليف کا سامنا ہے ؟*

*** کوئى درد/تکليف نہيں ہے  تھوڑے درد/تکليف کا سامنا ہے  شديد درد/تکليف کا سامنا ہے***

**61.4***۔ کيا بچہ اُداس / فکرمند ہے؟*

*** اُداس/فکرمند نہيں ہے  تھوڑا اُداس/فکرمند ہے  بہت اُداس/فکرمند ہے***

**62ـ علاج کا دورانيہ ختم ہونے کے بعد کا ردعمل**

**62.1***۔ جن شکايات کے ساتھـ آيا ہے (نشانات اور علامات)؟*

*** ٹھيک ہوگئيں  کچھـ ٹھيک ہوئيں  ٹھيک نہيں ہوئيں***

**62.2***۔ علاج کا نتيجہ؟*

*** علاج مکمل ہوگيا  علاج کيلئے واپس نہيں آيا  علاج ناکام رہا  مريض کى وفات  شمارنہيں ہوا***
